# Fe–Mn Biochar Composites from Sugarcane Bagasse for Herbicides Removal: Structure, Mechanisms, and Safety Relationships toward Sustainable Water Treatment

**DOI:** 10.1021/acsomega.5c12317

**Published:** 2026-01-29

**Authors:** Thamiris Ferreira Souza, Lucas Ferreira Fernandes, Laura Maria da Silva Batista, Carlos Henrique Milagres Ribeiro, Fábio Rodrigo Piovezani Rocha, Rafael Pio, Guilherme Max Dias Ferreira

**Affiliations:** † Group of Materials, Interfaces, and Solutions (MatIS), Department of Chemistry, 67739 Federal University of Lavras, Campus Universitário, P.O. Box 3037, Lavras, Minas Gerais 37200-000, Brazil; ‡ Department of Agriculture, Federal University of Lavras, Campus Universitário, P.O. Box 3037, Lavras, Minas Gerais 37200-000, Brazil; § Center for Nuclear Energy in Agriculture (CENA), University of São Paulo, Av. Centenário, 303, Piracicaba, São Paulo 13416-000, Brazil

## Abstract

Fe–Mn biochar composites were synthesized from sugarcane bagasse through prepyrolytic impregnation with FeCl_3_ and MnCl_2_, using immersion (IME) and coprecipitation (COP) methods, followed by pyrolysis at 600 °C for 2 h. Their characterization revealed distinct differences in surface chemistry and oxide dispersion. Both composites contained mixed Fe_3_O_4_, Fe_2_O_3_, MnO, and Mn_3_O_4_ phases, but IME exhibited a amorphous carbon matrix, while COP displayed greater crystallinity (∼41%). In aqueous adsorption studies, IME maintained nearly constant removal efficiency across pH 2–10, whereas COP was strongly pH-dependent, leading to IME’s selection for subsequent studies. Adsorption isotherms of 2,4-dichlorophenoxyacetic acid (2,4-D) and picloram (25 °C; 2 g L^–1^) were well fitted by the Sips model, with maximum adsorption capacities of 18.1 and 8.1 mg g^–1^, respectively. X-ray photoelectron spectroscopy of IME revealed Fe^3+^/Fe^2+^ and Mn^3+^/Mn^2+^ species and indicated that 2,4-D removal occurred mainly by Fe^3+^-carboxylate complexation, while picloram adsorption involved weaker polar and van der Waals interactions. Reuse tests showed a decline in performance after three cycles (∼97% → 29%), suggesting active-site blockage. Metal leaching from IME at pH 5 was limited (0.025 mg L^–1^ for Fe and 2.94 mg L^–1^ for Mn). Fe complied with drinking-water limits, whereas Mn exceeded them, highlighting the need for safety evaluation. Phytotoxicity assays using *Cucumis sativus* confirmed no adverse effects from residual 2,4-D, demonstrating effective detoxification. Overall, Fe–Mn biochar composites present a promising, sustainable approach for herbicide removal, but the environmental safety of treated effluents should be ensured.

## Graphical abstract

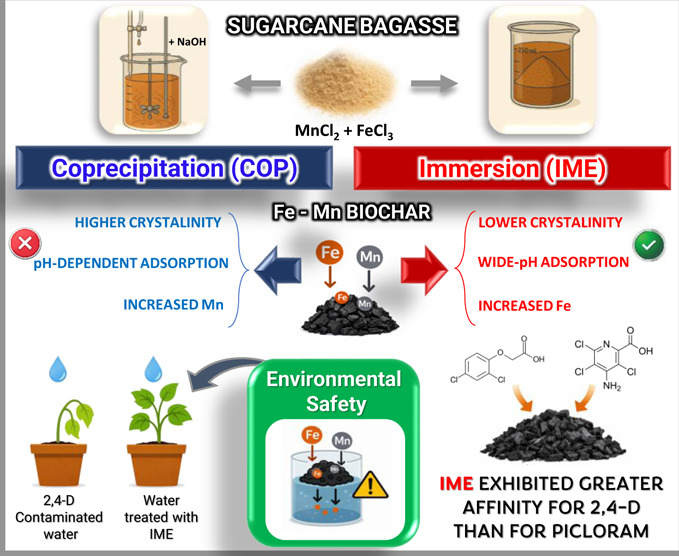

## 1 Introduction

The intensive use of synthetic herbicides in modern agriculture, although contributing to increase crop productivity,^1^ can result in the contamination of natural resources. Contamination of aquatic environments by these compounds is a public health concern,^2^ which impacts environmental health, and causes adverse effects in nontarget organisms, including carcinogenicity^3^ and endocrine disruption^4^ in humans and animals. Furthermore, the use of contaminated irrigation water containing such herbicides can harm subsequent crops due to the carryover phenomenon, leading to additional crop damage and agricultural losses.

2,4-Dichlorophenoxyacetic acid (2,4-D) was one of the first synthetic herbicides to be commercialized and is widely used in agricultural areas for the selective control of invasive plants.^5^ Picloram (4-amino-3,5,6-trichloro-2-pyridinecarboxylic acid), in turn, is a highly persistent pyridine herbicide.^2^ Despite their distinct properties (Table S1), both act as synthetic auxins, mimicking plant hormones and interfering with the development of target organisms.^6,7^ Picloram is frequently combined with 2,4-D in the Grazon P + D formulation (10.2% picloram and 39.6% 2,4-D), expanding the spectrum of broadleaf weed control.^8^

Given the high persistence and environmental impacts of herbicides, biochar-based materials have emerged as a promising alternative for their removal from contaminated waters.^9–11^ Biochar produced from agricultural residues such as sugarcane bagasse (SGB) can show porous structure, high surface area, and versatile surface chemistry, making it an attractive material for the development of novel adsorbents while simultaneously contributing to sustainable waste management.^10^ However, unmodified biochar often shows limited capacity for the removal of organic contaminants such as herbicides,^9^ prompting the development of strategies involving its surface modification.

The incorporation of transition metal oxides, such as those of Fe and Mn, into biochar, either in monometallic^12–14^ or bimetallic^15–17^ forms, has yielded composites with synergistic properties, leading to more efficient removal mechanisms and enhanced adsorption capacity compared with unmodified materials, as highlighted in recent critical reviews on metal-modified biochars.^13,18–20^ Most studies in this field have predominantly focused on metal adsorption, largely attributed to the formation of inner–sphere complexes between adsorbates and adsorbents.^12,13^ Reports focusing on the removal of organic contaminants such as pharmaceuticals^15,21^ and dyes^22^ using biochar composites containing Fe, Mn, or Fe–Mn based oxides have also demonstrated marked improvements in adsorption performance. With respect to 2,4-D, current investigations remain largely restricted to monometallic biochar systems,^23–27^ which is often referred to as activated carbon. In contrast, the adsorption of picloram using mono- or bimetallic biochar-metal oxide composites has not yet been addressed, representing an open avenue for exploration.

The production route plays a critical role in defining the crystalline structure, surface composition, and spatial distribution of metal oxides in the biochar composites. The prepyrolysis modification is advantageous due to the possibility of incorporating metals to produce the desired oxides simultaneously with the carbonization process. This one-step procedure saves time, reduces costs, and increases operational efficiency. In immersion methods, metal incorporation proceeds gradually, often leading to heterogeneous oxide dispersion and variable crystallite sizes after pyrolysis.^28^ Conversely, coprecipitation, often carried out under alkaline conditions, favors simultaneous nucleation and controlled growth of metallic particles on the carbonaceous matrix, promoting a more homogeneous distribution of oxides and the formation of specific crystalline phases.^29^

The selection of Fe–Mn bimetallic system for producing biochar composites is driven not only by the abundance and low cost of their precursors but also by their reported ability to improve material stability, particularly under acidic conditions.^23,30,31^ Based on these previous studies, it is hypothesized that the incorporation of Fe alongside Mn can mitigate metal leaching compared with monometallic modified biochars. Moreover, the coexistence of Mn and Fe oxides may increase the diversity of adsorption sites, potentially enhancing herbicide removal while improving the environmental stability of the composite materials. This is a crucial advantage, as metal leaching can introduce secondary contaminants, compromising both the safety and the sustainability of the adsorbent. Within this context, assessing the phytotoxicity of treated effluents is essential for risk evaluation in agricultural and ecological settings involving reuse of treated waters.

This study leverages Fe–Mn incorporation strategies to address a frequently overlooked issue: the environmental risks associated with metal-impregnated biochar composites, with an emphasis on scenarios involving water reuse and agricultural applications. Particularly, the study aimed to (i) investigate, for the first time, the adsorption of 2,4-D and picloram herbicides on Fe–Mn oxide-impregnated biochar composites, prepared by prepyrolysis modification routes using SGB; (ii) compare two incorporation methods of Fe–Mn based oxides, immersion and coprecipitation, in relation to the structural, chemical, and adsorptive properties of the resulting materials; and (iii) assess the environmental safety of these composites through leaching and phytotoxicity assays. To this end, characterization techniques were employed to elucidate surface chemistry and adsorption mechanisms, while batch experiments and kinetic/isotherm modeling were used to evaluate the performance and removal mechanisms of the herbicides. The study contributes to the development of safer and more efficient biochar-based adsorbents for herbicide remediation in water as well as supporting strategies for the sustainable management of agro-industrial residues.

## 2 Materials and Methods

### 2.1 Chemicals

The reagents used in this study included FeCl_3_ (98%, Exodus Scientific), MnCl_2_·4H_2_O (99.5%, Exodus Scientific), NaOH (98%, Synth), NaCl (99.5%, Quimica Moderna), HCl (36.46%, Synth), 2,4-dichlorophenoxyacetic acid (97%, Sigma-Aldrich), and picloram (99%, Sigma-Aldrich), all analytical grade. Deionized water was used for the preparation of all aqueous solutions. SGB from a sugar and ethanol plant in São Paulo, Brazil, was used to produce the biochars.

### 2.2 Biochar Preparation

Briefly, the SGB was dried, ground, and sieved (40 mesh) before undergoing bimetallic modification with a mixed FeCl_3_/MnCl_2_ solution (0.125 mol L^–1^ of each metal salt), concentration chosen based on a previous study.^23,27^ Two modification methods were applied to the biomass: immersion (to generate the IME material) and coprecipitation (to generate the COP material). In the former, SGB was immersed in the salt solution for 24 h at room temperature. In the coprecipitation method, SGB was dispersed in the metal salt solution and mechanically stirred using an Ultra-Turrax homogenizer, while NaOH (1 mol L^–1^) was added dropwise until the pH reached ∼12. For both treatments, the supernatant was separated from the treated biomass, which was then dried at 60 °C until a constant weight.

The dried solids were carbonized in a muffle furnace (EDG, 3000 3P, Brazil) at 600 °C (10 °C min^–1^) for 2 h. The same process was applied to unmodified SGB for comparison (BCS material). After cooling, the biochars were sequentially washed with 0.01 M HCl, 0.01 M NaOH, and water until neutral pH, dried, sieved (100 mesh), and stored in a desiccator.

### 2.3 Biochar Characterization

Before adsorption, the materials were characterized by scanning electron microscopy (SEM) and energy-dispersive X-ray spectroscopy (EDS), using a Tescan Clara-UHR ultra-high-resolution scanning electron microscope (Czech Republic) equipped with an EDS system (Bruker-Quantax, USA). Fourier transform infrared (FTIR) spectra were obtained by using a Varian Series 600-IR FT-IR spectrometer (USA) in the attenuated total reflectance mode. The number of acidic (*n*_af_) and basic (*n*_bf_) functions was determined by conductometric titration using an MS-Tecnopon conductivity meter (Brazil). The thermal stability of the materials was evaluated by using a DTG-60 AH thermogravimetric analyzer (Shimadzu, Japan). The point of zero charge (pH_PZC_) was determined using the solid addition method. X-ray diffraction (XRD) analyses were performed using a D2 Phaser diffractometer (Bruker, USA). The detailed analytical methodologies was previously described by Souza *et al.* ^23^

X-ray photoelectron spectroscopy (XPS) was performed for IME and COP materials using a Κ-alpha XPS spectrometer (Thermo Scientific, USA) equipped with a monochromatic source and Al anode, with a Kα energy of 1486 eV. For the survey spectra acquisition, the experimental conditions used were 10 scans, spot size of 300 μm, pass energy of 150.0 eV, energy step size of 1.000 eV, and dwell time of 10 ms. For high-resolution spectra acquisition, the conditions were adjusted to 10 scans, a spot size of 300 μm, a pass energy of 50.0 eV, an energy step size of 0.10 eV, and a dwell time of 50 ms. High-resolution analyses were performed specifically for C, O, Mn, and Fe to obtain detailed information about the chemical states and bonding configurations of these elements. For IME, the analyses were performed before and after the adsorption of 2,4-D and picloram (initial concentration of 400.0 mg L^–1^, 2.00 g L^–1^ of adsorbent, 24 h of contact, without pH adjustment: initial pH values of 5 and 4 for 2,4-D and picloram, respectively)

### 2.4 Single-Component Batch Adsorption Experiments

The systems for adsorption studies were prepared in glass vials and maintained in an incubator shaker (NT715, Nova Técnica, Brazil) at 120 rpm and 25 ± 1 °C throughout the adsorption process. After the selected contact time, the supernatants were collected, centrifuged at 3200 rpm for 5 min, and analyzed by molecular absorption spectrophotometry (UV–vis AJ Micronal model AJX-3000PC, Brazil) at 284 nm for 2,4-D and 223 nm for picloram.

The analytical method for 2,4-D analysis showed limits of detection (LODs) of 0.02 mg L^–1^ and 0.15 mg L^–1^ for IME and COP, respectively. For picloram, the LODs were 0.002 mg L^–1^ and 0.009 mg L^–1^ for IME and COP, respectively. The remaining concentrations after adsorption were determined using analytical curves, which were linear in the range 5–40 mg L^–1^ for 2,4-D (slope: 0.0088; intercept: 0.0023; *R* ^2^: 0.9992) and 3 to 25 mg L^–1^ for picloram (slope: 0.1336; intercept: 0.0111; *R* ^2^: 0.9994).

The amount adsorbed at equilibrium (*q*_e_, mg g^–1^) and the percentage removal (% *R*) of the herbicides were calculated according to eqs 1 and 2, respectively.

1 $$q_{e}=\frac{\left(C_{i}-C_{e}\right)\cdot V}{m}$$

2 $$\%\;R=\frac{C_{i}-C_{e}}{C_{i}}.100$$

where *C*_*i*_ and *C*_e_ are the initial and equilibrium concentrations (mg L^–1^), respectively, *m* is the mass of the adsorbent (g), and *V* is the volume of the solution (L).

A preliminary adsorption screening was carried out using BCS, COP, and IME under the following conditions: 20.0 mg L^–1^ of 2,4-D or picloram, 2.00 g L^–1^ of adsorbent, and 24 h of contact. The adsorption on IME and COP was further examined by assessing: (i) the effect of initial pH (2–12) under the same contaminant and adsorbent concentrations and 24 h of contact and (ii) the effect of NaCl concentration, adjusted from 0 to 0.100 mol L^–1^, under the previously described experimental conditions.

Subsequent experiments focused exclusively on IME were performed at 25 ± 1 °C, investigating: (i) adsorption kinetics over contact times ranging from 10 to 1800 min (20.0 mg L^–1^ of 2,4-D or picloram, 2.00 g L^–1^ of composite); (ii) adsorption isotherms constructed by varying the initial concentration of the adsorbate in the range of 10–400 mg L^–1^ (24 h of contact, 2.00 g L^–1^ of composite); and (iii) the effect of adsorbent dose (1, 2, 4, and 8 g L^–1^, 24 h of contact). All experiments were conducted in duplicate without pH adjustment, with blank tests performed in the absence of the herbicides for each condition.

To describe the adsorption kinetics, nonlinear regression models, including pseudo-first-order (PFO),^32^ pseudo-second-order (PSO),^33^ Elovich,^34^ and intraparticle diffusion^35^ models, were applied. Langmuir,^36^ Freundlich,^37^ and Sips^38^ models were used to fit the adsorption isotherm data. All model fittings were performed using nonlinear regression analysis in OriginPro 9.1 software. The adjust to the models was evaluated by the determination coefficient (*R* ^2^), residual sum of squares (RSS), and Akaike Information Criterion (AIC), which allows for the relative comparison of the statistical models considering simultaneously the goodness of fit and model complexity.^39^ Additional details on the adsorption models are provided in Table S2 (Supporting Information).

### 2.5 Reusability of Adsorbent

The reuse of IME in adsorption/desorption experiments was evaluated with 0.0600 g of adsorbent added to an Erlenmeyer flask containing 30.00 mL of a 10.0 mg L^–1^ solution of either 2,4-D (pH 5) or picloram (pH 4). The mixture was agitated for 24 h at 25 ± 1 °C and 120 rpm, followed by centrifugation at 3000 rpm for 5 min. After the supernatant was removed, the adsorption capacity was determined as described in Section 2.4. For regeneration, the material was treated with 10.00 mL of 0.01 mol L^–1^ NaOH solution for 2 h under the same temperature and agitation conditions, rinsed repeatedly with deionized water until reaching neutral pH, and subsequently dried at 60 °C. The regenerated adsorbent was subjected to three additional adsorption/desorption cycles. All experiments were conducted in triplicate with appropriate blank evaluation.

### 2.6 Composition of Fe–Mn Biochar Composites and Metal Leaching

The IME and COP materials, before and after contact with aqueous solutions adjusted to pH 2, 7, or 12 (using HCl or NaOH solutions), were subjected to microwave-assisted acid digestion in triplicate. For each assay, 100 mg of sample was placed in decomposition vessels, followed by the addition of 6 mL of HNO_3_ (20% v v^–1^) and 2 mL of H_2_O_2_ (30% v v^–1^) (Merck, Germany). The mixtures were heated at 160–230 °C in a microwave system equipped with a high-pressure reactor (UltraWAVE MCLA 1000–60, Milestone, Italy). After complete digestion, the solutions were diluted to a final volume of 30.00 mL with deionized water. Blank solutions were prepared under the same conditions, with the sample replaced with deionized water.

The determination of total mineral content was performed using inductively coupled plasma optical emission spectrometry (ICP-OES, iCAP 7000, Thermo Scientific, USA) with a radial view. The operating parameters were as follows: radiofrequency power of 1.2 kW, plasma gas flow of 12 L min^–1^, auxiliary gas flow of 0.5 L min^–1^, nebulizer gas flow of 0.6 L min^–1^, and sample aspiration rate of 1.5 mL min^–1^. Data acquisition and processing were carried out using the Qtegra Intelligent Scientific Data Solution software (Thermo Scientific). The monitored emission lines were: Na—589.592 nm; Mg—279.553 nm; Al—396.152 nm; Ca—396.847 nm; Mn—259.373 nm; Fe—259.940 nm; Cu—327.396 nm; and Zn—213.856 nm.

Leaching of Fe and Mn from the biochars after 2,4-D adsorption was evaluated in the following experiments: the effect of initial pH (COP and IME), the effect of adsorbent dose (IME), and adsorbent reuse (IME). After adsorption and centrifugation (as described in Section 2.4), the supernatants were collected for both determination of the remaining herbicide amount and leached Fe and Mn. The latter was based on flame atomic absorption spectrometry (FAAS, Shimadzu AA-700, Japan), performed under manufacturer recommendations and measurements wavelengths of 248.3 nm (Fe) and 279.5 nm (Mn).

### 2.7 Phytotoxicity Tests

Phytotoxicity was evaluated by a seed germination bioassay adapted from Santos *et al*.^40^ and seed analysis rules of Brazil,^41^ employing *Cucumis sativus* seeds (Esmeralda type, caipira variety, Lot 0008802310000080, Feltrin Sementes, Brazil). Four treatments were applied: deionized water (T1), 5 mg L^–1^ 2,4-D solution (T2), 5 mg L^–1^ 2,4-D solution after treatment with IME (T3), and solution containing only water after contact with IME in the absence of 2,4-D (T4), to evaluate the possible leaching of compounds from the material. For T3 and T4, samples were agitated in an incubator shaker (NT715, Nova Técnica, Brazil) at 120 rpm for 24 h at 25 ± 1 °C, and solids were separated by centrifugation (5000 rpm, 5 min) prior to seed exposure.

The assays were conducted in Petri dishes lined with filter paper (85 g m^–2^, Ø 11.0 cm), with ten seeds placed per dish, and 5 mL of each solution (treatment T1–T4). The dishes were incubated in a B.O.D. climatic chamber (Eletrolab, Brazil) for 8 days at 25 ± 1 °C, under a photoperiod of 8 h of light and 16 h of dark. Germination was assessed on day 3 and at the end of the assay by evaluating: number of leaves, shoot height and diameter, root length, fresh weight, and dry weight (after drying at 40 °C for 48 h). The assays were conducted in quadruplicate. Data were analyzed by ANOVA and multiple comparisons using Tukey’s test, with the analyses performed using RStudio software (v. 4.2.2)^42^ and the ExpDes package.^43^

## 3 Results and Discussion

### 3.1 Biochar Characterization

#### 3.1.1 Surface Functional Groups and Chemical Analysis of the Biochars

FTIR spectroscopy was employed to investigate the surface functional groups of BCS and Fe–Mn biochar composites (Figure 1a,b). A detailed assignment of the absorption bands is provided in Table S3 of the Supporting Information.

**Figure 1:**
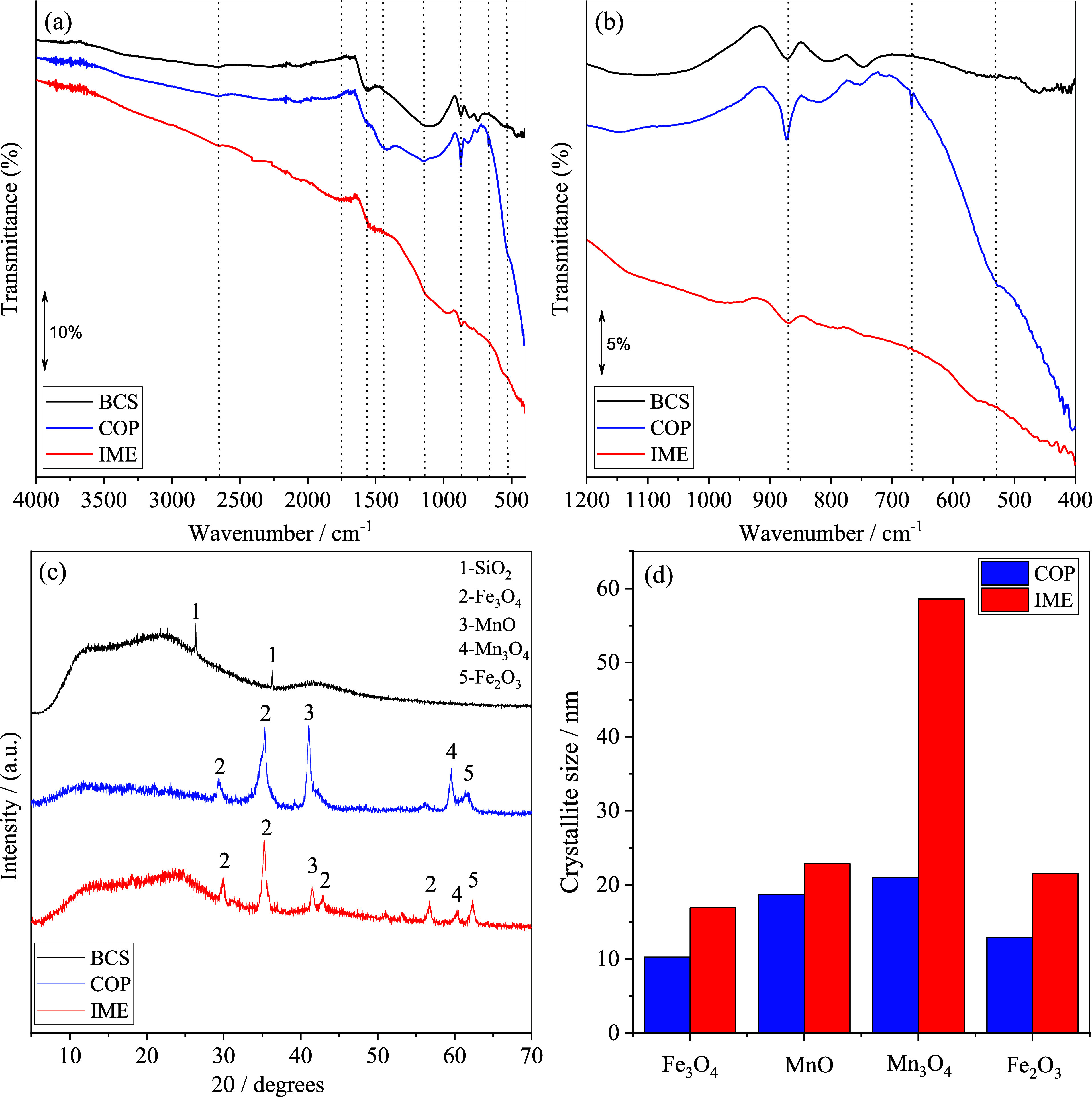
(a) FTIR spectra; (b) enlarged view of the 1200–400 cm^–1^ FTIR region; (c) XRD patterns; (d) crystallite sizes of the identified phases in the composites.

The spectra display low-intensity bands, with only subtle variations among the samples. This behavior is consistent with the high degree of carbonization at 600 °C, which markedly reduces the abundance of functional groups originally present in the biomass.^44^ The absence of the band at ∼3500 cm^–1^ reflects the reduction of surface hydroxyl groups caused by thermal treatment and metal oxide incorporation during biochar production. The most evident differences among the three materials were observed in the 1200–400 cm^–1^ region (Figure 1b). A similar pattern was reported by Lin *et al*.^45^ for Fe–Mn oxide-impregnated corn stalk biochars obtained by pyrolysis at 620 °C for 3 h.

Despite the absence of bands in the 2850–2960 cm^–1^ region, typically associated with symmetric and asymmetric stretching of aliphatic –CH_2_ and –CH_3_ groups,^46^ the IME sample exhibited a band at 1440 cm^–1^ (aliphatic –CH_2_ bending), suggesting partial preservation of aliphatic structures in this material. In contrast, bands related to aromaticity were more evident: the C=C stretching band (∼1564 cm^–1^) appeared in both BCS and IME, shifting to 1530 cm^–1^ in COP,^47^ while the out-of-plane C–H bending at 873 cm^–1^ ^48^ showed higher intensity in all samples. Additionally, a weaker band at 669 cm^–1^, also attributed to aromatic C–H out-of-plane vibrations, was evidenced in COP. These features indicate increased aromatic character of the composites due to carbonization with IME retaining some aliphatic moieties.

The band at approximately 1750 cm^–1^ in the IME material (with a very low intensity for COP) indicates carboxylic groups. The C–O–C stretching band at 1139 cm^–1^ was more pronounced in IME in comparison to BCS, and nearly absent in COP. In addition, subtle bands at 527 cm^–1^ (IME) and 564 cm^–1^ (COP) were assigned to Fe–O and Mn–O stretching, respectively. These spectral features show the modification routes distinctly altered the surface chemistry of the Fe–Mn modified biochars.^49^

Conductimetric titration (Figure S2 and Table S4) revealed that the Fe–Mn incorporation method (immersion or coprecipitation) significantly altered the total amount of acidic and basic functional groups. The BCS sample presented a total functional group content (*n*_af_ + *n*_bf_) of 2.25 mmol g^–1^, consisting of 1.63 mmol g^–1^ acidic groups and 0.62 mmol g^–1^ basic groups. In IME, the total functional group content increased to 4.49 mmol g^–1^, dominated by acidic groups (*n*_fa_ = 3.06 mmol g^–1^ versus *n*_fb_ = 1.43 mmol g^–1^). Conversely, COP exhibited the highest *n*_af_ + *n*_bf_ (6.68 mmol g^–1^), with the predominance of basic groups (*n*_bf_ = 3.98 mmol g^–1^ and *n*_af_ = 2.70 mmol g^–1^). These differences can be attributed to the distinct chemical processes employed in the modification methods. In BCS, the functional composition reflects the original biomass structure and carbonization process. For IME, the direct interaction between the incorporated metals and the biomass functional groups likely promoted the stabilization or formation of acidic sites during pyrolysis. During thermal treatment, the metals likely formed stable metal–oxygen surface complexes with the biomass, which acted as Lewis acidic sites.^50^ Additionally, Fe and Mn may have catalyzed oxidation and rearrangement reactions, promoting the formation and stabilization of oxygenated functions (e.g., carboxylic groups), corroborating the FTIR results. In contrast, for COP, precipitation with NaOH favored the generation of basic groups by neutralizing soluble acidic compounds and incorporating hydroxyl-rich phases. Besides, formation of metal hydroxides in the prepyrolysis treatment prevented the metal to interact effectively with functional groups on the biomass and catalyze the formation of acidic groups.^51^

The pH_PZC_ values (Figure S3 and Table S4) for BCS, IME, and COP were 8.05, 4.21, and 9.6, respectively, reflecting the functional group composition. Previous studies have indicated that these differences are not exclusively associated with the modification method but rather with other process variables. Lin *et al*.^45^ reported a pH_PZC_ of 9.80 for a Fe–Mn-biochar-modified postpyrolysis with KMnO_4_ and Fe­(NO_3_)_3_ (corncob, 620 °C, 3 h) using immersion method. In contrast, Wang *et al*.^52^ observed a pH_PZC_ of 4.16 for a Fe–Mn biochar-modified prepyrolysis with KMnO_4_ and FeCl_3_ (aquatic plant cattail, 500 °C, 4 h) using coprecipitation with NaOH. Lin *et al*.^53^ produced a biochar from corn straw (600 °C, 2 h) and modified it by immersion in KMnO_4_ and Fe­(NO_3_)_3_ solutions, obtaining a pH_PZC_ of 9.6. However, when Fe­(NO_3_)_3_ was replaced with FeSO_4_ in the immersion process, the pH_PZC_ was significantly reduced to 3.17. These results highlight the influence of the type of metal salt (or even the type of biomass) on the chemical modification of the biochar surface, demonstrating that different metallic precursors can induce substantial variations in the acid–base properties of the material.

#### 3.1.2 Crystalline Properties

The diffractograms of the materials are shown in Figure 1c. For BCS, the peaks at 2θ = 22.74° and 41.59° correspond to SiO_2_ (α-quartz; COD-ID 8103513),^54^ although the sample is predominantly amorphous with less than 1% crystallinity index, calculated as the ratio of the crystalline area to the total area of the XRD diffractograms. The diffraction peaks identified for IME and COP correspond to the crystalline phases of Fe_3_O_4_ (Magnetite; COD-ID 9005837), Fe_2_O_3_ (Hematite; COD-ID 1011240), MnO (Manganosite; COD-ID 1514099), and Mn_3_O_4_ (Hausmannite; COD-ID 9001963). The average crystallite sizes, determined from the four peaks with the highest full width at half-maximum, were 21 ± 3 nm for IME and 16 ± 5 nm for COP (Tables S5 and S6), indicating that the oxides are present at the nanometer scale.^55^

Previous investigations of Fe–Mn biochar composites show that different synthesis conditions influence the formation and distribution of the crystalline phases of iron and manganese oxides. Zhou *et al*.^56^ also developed bimetallic biochars from sugarcane bagasse, enriched with Fe and Mn by impregnation followed by pyrolysis at 750 °C for 1 h, using FeCl_2_·4H_2_O and (CH_3_COO)_2_Mn·4H_2_O as precursors. In the Fe:Mn molar ratio of 1:1 composite, Fe_3_O_4_ and Mn_3_O_4_ phases predominated, while in the 2:1 ratio, Fe_2_O_3_ and Fe_3_O_4_ were identified, with no significant peaks of Mn_3_O_4_. Similarly, Liang *et al*.^57^ produced a biochar from branches under pyrolysis at 800 °C for 2 h and postpyrolysis impregnation with FeCl_3_ and MnCl_2_ in a 4:1 ratio, followed by calcination at 800 °C for 1 h. The XRD analysis revealed the presence of Fe_3_O_4_, while the characteristic peaks of Mn oxides were not detected, possibly due to the low loading of these oxides in the material.

In the present study, Fe_3_O_4_ peaks predominate in both materials, especially in IME. During the dissolution of FeCl_3_ and MnCl_2_ and their mixture with the biomass in the immersion approach, the pH between 3 and 4 favors the precipitation of Fe­(OH)_3_ (eq 3), while Mn^2+^ remains dissolved in the solution or adsorbed on the biomass. During pyrolysis, residual oxygen in the system can promote the initial reaction of Fe­(OH)_3_ to Fe_2_O_3_ (eq 4) on the surface layers. Subsequently, the carbon from the biomass, formed at high temperatures, can reduce part of Fe_2_O_3_ to Fe_3_O_4_ (eq 5).^58–60^

3 $${Fe}^{3+}\left(aq\right)+{3OH}^{-}\left(aq\right){\to Fe\left(OH\right)}_{3}\left(s\right)$$

$${,2Fe\left(OH\right)}_{3}\left(s\right)\overset{\Delta }{\to }{Fe}_{2}O_{3}\left(s\right)+3H_{2}O\left(g\right)$$ 4

$${Fe}_{2}O_{3}\left(s\right)+C\left(s\right)\overset{\Delta }{\to }{2Fe}_{3}O_{4}\left(s\right)+CO\left(g\right)$$ 5

The higher relative intensity of Mn oxide peaks in COP compared to IME is consistent with the coprecipitation process, in which the biomass is pyrolyzed after Mn­(OH)_2_ formation (eq 6) under alkaline conditions (pH 12). In contrast, in IME, Mn^2+^ remains dissolved prior to pyrolysis, leading to lower availability of manganese in a preformed solid phase. During pyrolysis, Mn­(OH)_2_ is converted to Mn_3_O_4_ (eq 7),^61–63^ analogously to the conversion of Fe­(OH)_3_ into Fe_2_O_3_, with subsequent partial conversion of Mn_3_O_4_ into MnO (eq 8) in the reducing environment. As a result, the materials contain a mixture of Fe and Mn oxide phases.

6 $${Mn}^{2+}\left(aq\right)+2{OH}^{-}\left(aq\right){\to Mn\left(OH\right)}_{2}\left(s\right)$$

$${,3Mn\left(OH\right)}_{2}(s)\overset{\Delta }{\to }{Mn}_{3}O_{4}\left(s\right)+H_{2}\left(g\right)+2H_{2}O\left(g\right)$$ 7

$${Mn}_{3}O_{4}\left(s\right)+C\left(s\right)\overset{\Delta }{\to }3MnO\left(s\right)+CO\left(g\right)$$ 8

Although COP and IME share similar crystalline phases, there are significant differences in the intensities of Fe and Mn oxide peaks. The crystallinity index was approximately 41% for COP and 12% for IME. Analyzing the crystallite sizes of the individual oxides, it is observed that COP has smaller crystallites, especially for Mn_3_O_4_, whose crystallite size is almost three times smaller than that in IME (Figure 1d).

The results suggest that the coprecipitation method promoted rapid and homogeneous nucleation, which limited crystallite growth during pyrolysis, resulting in numerous small crystallites. In contrast, the immersion method led to a heterogeneous distribution of metals, yielding regions with varying precursor concentrations that favored the formation of fewer but larger, crystallites. Consequently, IME exhibits lower overall crystallinity due to the presence of amorphous phases interspersed with larger crystals. This distribution is further illustrated in the micrographs presented in the following section.

#### 3.1.3 Morphological Analysis and Chemical Characterization

The morphological and compositional properties of BCS, IME, and COP were evaluated by using SEM (Figure 2) and EDS (Figure S4 and Table S7). The micrographs revealed a carbonized structure with the presence of channels, especially in BCS, suggesting partial preservation of the original biomass’ structural organization.^64^ However, evidence of fiber fusion was observed after the pyrolysis process. The images also showed structures with varied morphologies, indicating differences in fragmentation and surface topography of the particles, possibly resulting from mechanical fracture during maceration and sieving, while smoother surfaces could be associated with fusion.

**Figure 2:**
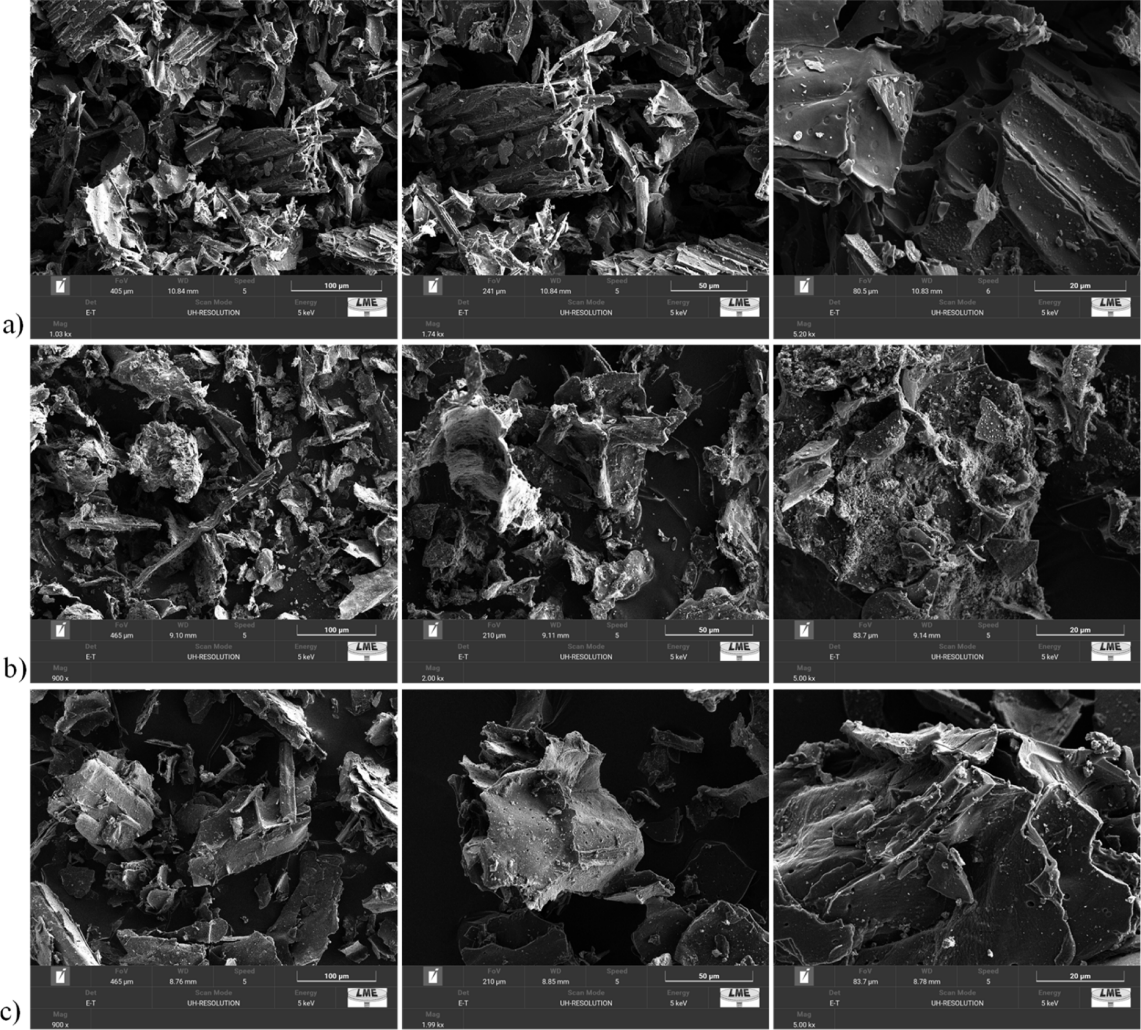
SEM images of (a) BCS, (b) COP, and (c) IME.

The Fe–Mn biochar composites displayed clear structural differences. IME closely resembled BCS in surface roughness but contained discrete microparticles of iron and manganese oxides,^65^ as confirmed by EDS mapping (see discussion below). In BCS, the observed granules corresponded to biomass-derived compounds, such as residual minerals or pyrolysis ash. In IME, the oxide crystallites were more dispersed, generating amorphous regions interspersed with crystalline domains. Conversely, COP exhibited greater roughness due to the formation of microparticle agglomerates, indicating a more extensive oxide deposition.^66^ These structural features are consistent with the XRD results and the oxide formation mechanisms previously discussed.

The EDS results (Figure S4) revealed the surface elemental composition of the materials. In BCS, oxygen (73.17%), calcium (13.05%), potassium (9.65%), and silicon (3.79%) predominate, reflecting residual minerals from the biomass, while the detection of chlorine (0.34%) indicates minor contamination during preparation. In IME, the surface composition was dominated by iron (33.74%), oxygen (18.78%), and manganese (16.46%). A substantial amount of chlorine (26.39%) was also detected, consistent with the FeCl_3_ and MnCl_2_ precursors used in the process. Trace levels of calcium (1.45%), aluminum (1.45%), and silicon (1.19%) suggested residual biomass-derived minerals.

COP presented a composition broadly similar to that of IME but with higher proportions of iron (42.11%) and manganese (40.64%), indicating higher impregnation of these elements into the biochar. In this case, the oxygen content was lower (8.65%), and sodium (2.11%) was detected due to NaOH. Smaller amounts of calcium (4.28%), aluminum (1.17%), and silicon (1.05%) were also observed. Elemental composition of the materials corroborated the XPS results (Section 3.1.4).

Elemental analysis by ICP-OES also revealed marked compositional differences between COP and IME (Table S8). COP was particularly enriched in Mn (128.3 g kg^–1^) and Fe (141.4 mg kg^–1^), whereas IME contained significantly lower levels of both elements (20.2 g kg^–1^ Mn and 53.7 mg kg^–1^ Fe). The higher Mn and Fe contents in COP suggest a stronger potential for metal–herbicide interactions, particularly through complexation and catalytic pathways. By contrast, IME presented a comparatively lower metal amount, which may restrict its capacity for redox-driven processes but favors adsorption mechanisms less influenced by transition-metal catalysis.

Both materials also differed in the abundance of alkaline and alkaline-earth elements. COP exhibited higher concentrations of Na, Ca, and Mg, which may contribute to electrostatic interactions and cation exchange processes, thereby enhancing the adsorption performance. Conversely, IME contained lower levels of these cations, suggesting fewer sites for ion-exchange-driven removal. Similarly, trace metals such as Cu and Zn were found at higher levels in COP than in IME, potentially providing additional coordination sites for herbicide binding.

These results showed that the modification methods significantly impacted the materials’ surface, influencing the distribution and concentration of elements, especially Fe and Mn oxides.

#### 3.1.4 X-ray Photoelectron Spectroscopy

The variations in chemical composition and elemental valence of the elements on COP and IME surfaces were evaluated using XPS. The survey spectra (Figure S5) and the obtained data (Table S9) indicated a higher atomic carbon content in IME (85.26%–285.1 eV) compared with COP (78.09%–285.1 eV), while COP exhibited a higher oxygen content (16.36%–531.4 eV) than IME (11.5%–532.3 eV). Additionally, the atomic percentages of Fe (1.78%–711.3 eV) and Mn (2.54%–641.9 eV) in COP were slightly higher than those observed in IME (Fe 1.27%–711.8 eV; Mn 0.59%–642.1 eV), as corroborated by elemental determination by ICP-OES (Table S8). The COP material also showed traces of sodium (Na 1s, 1.23%–1071.7 eV) due to the use of NaOH, while chlorine (Cl 2p, 1.41%–199.1 eV) was detected in IME, derived from the precursors FeCl_3_ and MnCl_2_.

A detailed analysis of the high-resolution C 1s, Mn 2p, and Fe 2p spectra (Figure 3) was performed, and the elemental compositions obtained from peak deconvolution are summarized in Table S10. The C 1s region (Figure 3a,b) showed a predominance of sp^2^ and sp^3^ hybridized carbon (C=C/C–C, 284.5 eV) for both materials, indicative of aliphatic and aromatic or graphitic structures. COP presented a higher proportion of oxygenated groups at 286.1 and 288.2 eV (C–O–C and O=C–O),^67^ and the presence of a π–π* satellite peak (291.3 eV), which indicates electron delocalization in conjugated aromatic systems.^68^ IME also exhibited oxygenated groups at 286.1 and 288.2 eV (C–O–C and O=C–O), but with a higher content of C–C/C=C than that of COP and the presence of C–OH (285.8 eV).

**Figure 3:**
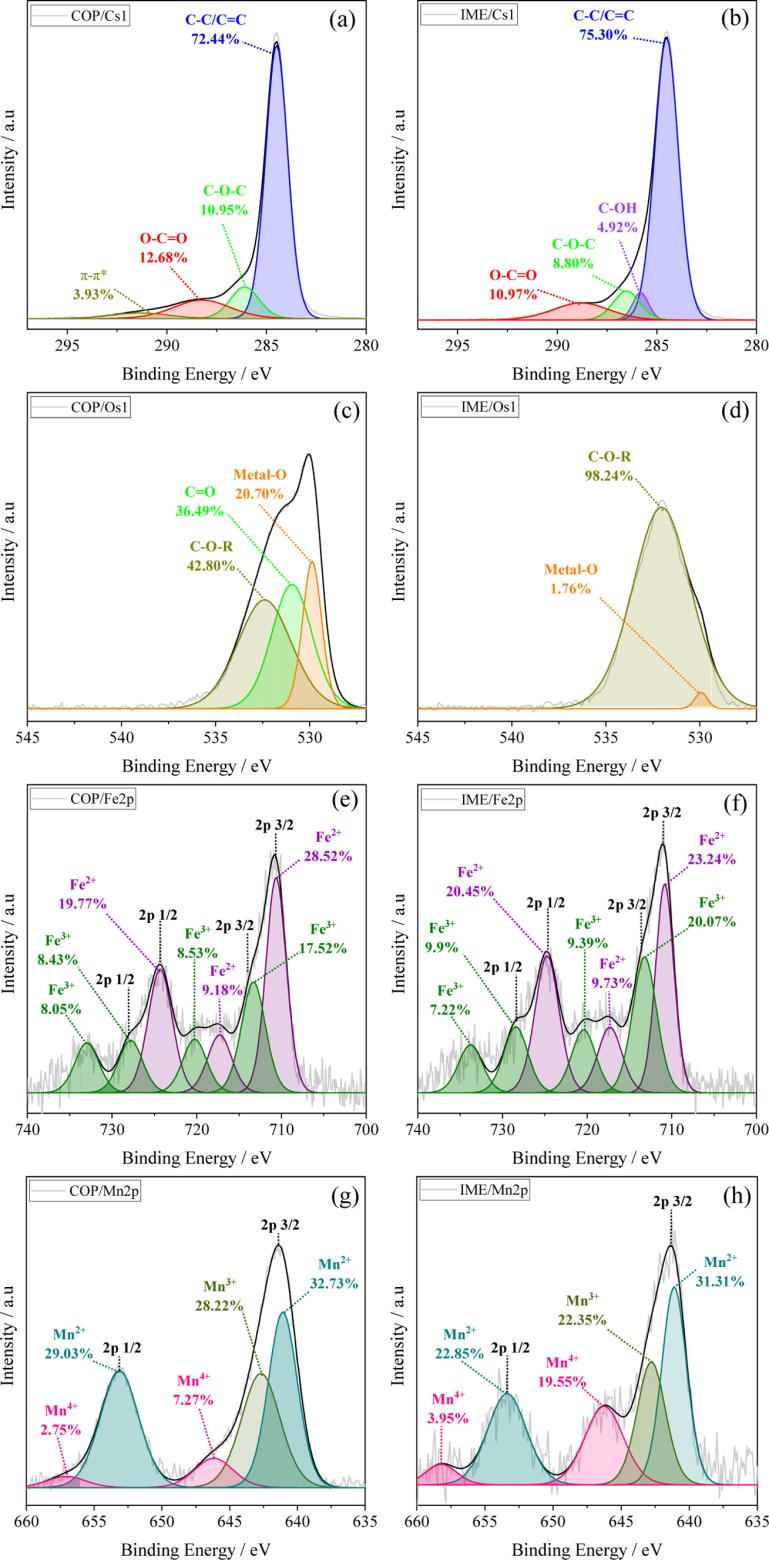
High-resolution C 1s (a,b), O 1s (c,d), Fe 2p (e,f), and Mn 2p (g,h) spectra with deconvolution for material COP and IME.

In the O 1s region (Figure 3c,d), IME was dominated by C–O–R groups (98.24% at 532.0 eV), with a minor contribution from metal–O bonds (1.76% at 529.9 eV). A distinct C=O peak (530.9 eV) was not detected, although the high-resolution C 1s spectrum revealed an O=C–O component, consistent with the FTIR band at ∼1750 cm^–1^. This band is related to protonated carboxylic acids, explaining why the O 1s deconvolution did not resolve a separate C=O contribution: the two nonequivalent oxygens in –COOH likely overlap with the C–O–R and metal–O signals. The predominance of protonated acidic groups on IME is further supported by its higher *n*_af_ value (Table S4). In contrast, COP showed a greater proportion of oxygen atoms bonded to metals with 20.70% Metal–O, 42.80% C=O (530.9 eV), and 36.49% C–O–R (532.4 eV).

The characterization of Fe species (Figure 3d,e) in the 2p_3/2_ and 2p_1/2_ orbitals revealed the coexistence of Fe^2+^ and Fe^3+^,^69^ with this mixed valence associated with oxides such as magnetite and hematite, corroborating XRD results. COP showed a higher proportion of Fe^2+^ (57.47%), while IME exhibited a more balanced distribution between Fe^2+^ (53.42%) and Fe^3+^ (46.58%). For Mn, in the 2p_3/2_ and 2p_1/2_ orbitals (Figure 3f–g), multiple oxidation states (Mn^2+^, Mn^3+^, and Mn^4+^) were also identified,^69^ in accordance with the presence of mixed oxides such as MnO, Mn_3_O_4_/Mn_2_O_3_, and MnO_2_. The absence of MnO_2_ peaks in the XRD diffractogram suggests its amorphous or dispersed form in the samples, with COP and IME predominantly containing Mn^2+^/Mn^3+^ and IME showing a higher amount of Mn^4+^ than COP.

The XPS results, together with conductometric titration and pH_PZC_ analyses, indicate that COP shows a more functionalized and oxidized surface, with a higher O/C ratio (0.21 compared to 0.13 for IME), enriched with oxygen-containing groups and metal-bound oxygen atoms. This composition gives COP a more hydrophilic character. In contrast, IME, with a lower O/C ratio, presents a less functionalized and more hydrophobic surface, which may favor interactions with weakly polar or nonpolar molecules.^65^ The persistence of such oxygenated groups after pyrolysis is likely due to stabilization by alkaline elements.^68^

The detection of the π–π* signal also suggests a more organized aromatic structure in COP. However, the agglomeration of metallic oxide microparticles observed by SEM/EDS indicates that these aromatic structures may have a lower availability for interactions. In contrast, IME presents a less functionalized and more amorphous surface, as evidenced by XRD. The carbonaceous area of IME, although less structured, is more accessible for interactions, as demonstrated by the SEM and EDS mapping images (Figures 2 and S4).

#### 3.1.5 Thermal Stability and Yield of Biochars

The thermal stability of the biomass and modified composites was evaluated through thermogravimetric analyses (Figures S6). The SB exhibited three decomposition stages of mass loss: 7% at 107 °C (loss of water and low-molecular-weight compounds^70^), 72% between 107 and 404 °C (decomposition of hemicellulose, cellulose, and part of the lignin^71^), and 21% between 404 and 900 °C (degradation of residual lignin and nonvolatile compounds^72^), leaving no significant residue (<1%). The immersion treatment (SB-IME) shifted the initial decomposition to 154 °C (20%), followed by mass losses of 37% (154–414 °C), 18% (414–586 °C), and 16% (586–900 °C), resulting in a final residue of 9%. The coprecipitation treatment with NaOH (SB–COP) increased the initial decomposition temperature to 138 °C, followed by losses of 32% (138–402 °C), 27% (402–730 °C), and 11% (730–900 °C), raising the residual fraction to 22%. As observed, the incorporation of Fe–Mn increased the thermal stability of the biomass, increasing the residual mass. The coprecipitation method intensified this effect, mainly due to the formation of Fe–Mn hydroxides prior to heating.

In biochars, thermogravimetric analysis (Figure S7) allowed for the assessment of the remaining lignocellulosic fraction after pyrolysis. BCS exhibited an initial mass loss of 5% at 391 °C, followed by successive losses of 14% (391–646 °C), 14% (646–768 °C), and 15% (768–900 °C), resulting in a final residue of 52%. IME showed an initial loss of 11% up to 407 °C, with subsequent losses of 7% (407–557 °C), 10% (557–703 °C), and 21% (703–900 °C), leading to a residual fraction of 51%. COP exhibited the lowest initial mass loss (5% up to 312 °C), followed by losses of 12% (312–537 °C), 18% (537–752 °C), and 19% (752–900 °C), resulting in the lowest final residue mass (47%).

The results indicate that the residual fraction of the adsorbents at 900 °C followed an inverse trend compared to modified biomasses, highlighting the combined impact of pyrolysis and metal oxide formation on the thermal stability of the materials. The lowest retention of fixed carbon was observed in BCS (indicated by the higher mass loss in the last three stages of degradation), suggesting greater thermal degradation of the carbonaceous matrix. Thus, biochar modification not only altered its thermal stability but also influenced its final composition due to the catalytic effect of the metals.^73,74^

These observations are consistent with the pyrolysis yields, which varied significantly among the treatments, with values of 23.41% for BCS, 40.90% for IME, and 51.01% for COP (Table S4). The increased yield in the modified biochars is not necessarily associated with the carbonaceous matrix but also with the incorporation of Fe and Mn oxides.^23^ Thus, the higher COP yield reflects its higher content of Fe and Mn oxides (Table S8).

### 3.2 Batch Adsorption Studies

#### 3.2.1 Preliminary Adsorption Test

In the preliminary adsorption tests (20.0 mg L^–1^ of 2,4-D or picloram; 2.00 g L^–1^ of adsorbent; unadjusted pH; 120 rpm; 25.0 °C), none of the materials achieved more than 50% removal for either contaminant. Removal was consistently higher for 2,4-D (Figure S8a) than for picloram (Figure S8b). Among the materials, IME exhibited the highest removal performance, followed by COP and then BCS. Although π–π interactions between the aromatic rings of the herbicides and the carbon framework of unmodified biochar can contribute to adsorption, previous studies have shown that this contribution is minor for biochars produced at low pyrolysis temperatures, mainly due to their limited specific surface area.^23^ In this context, several studies have emphasized the necessity of biochar modification to enhance herbicide removal efficiency.^9,75,76^

Interestingly, despite its higher content of metal, COP performed worse than IME. To clarify the influence of the modification method on herbicide adsorption, IME and COP were further investigated.

#### 3.2.2 Effect of Initial pH and Ionic Strength on 2,4-D and Picloram Adsorption

The initial pH of the solution plays a crucial role in contaminant adsorption as it affects both the surface charge of the adsorbent and protonation–deprotonation equilibria involving the adsorbates. As shown in Figure 4, the highest removal efficiencies for both herbicides were obtained at pH 2.0 (i.e., 98% for 2,4-D and 84% for picloram (COP) and 52% for 2,4-D and 34% for picloram (IME).

**Figure 4:**
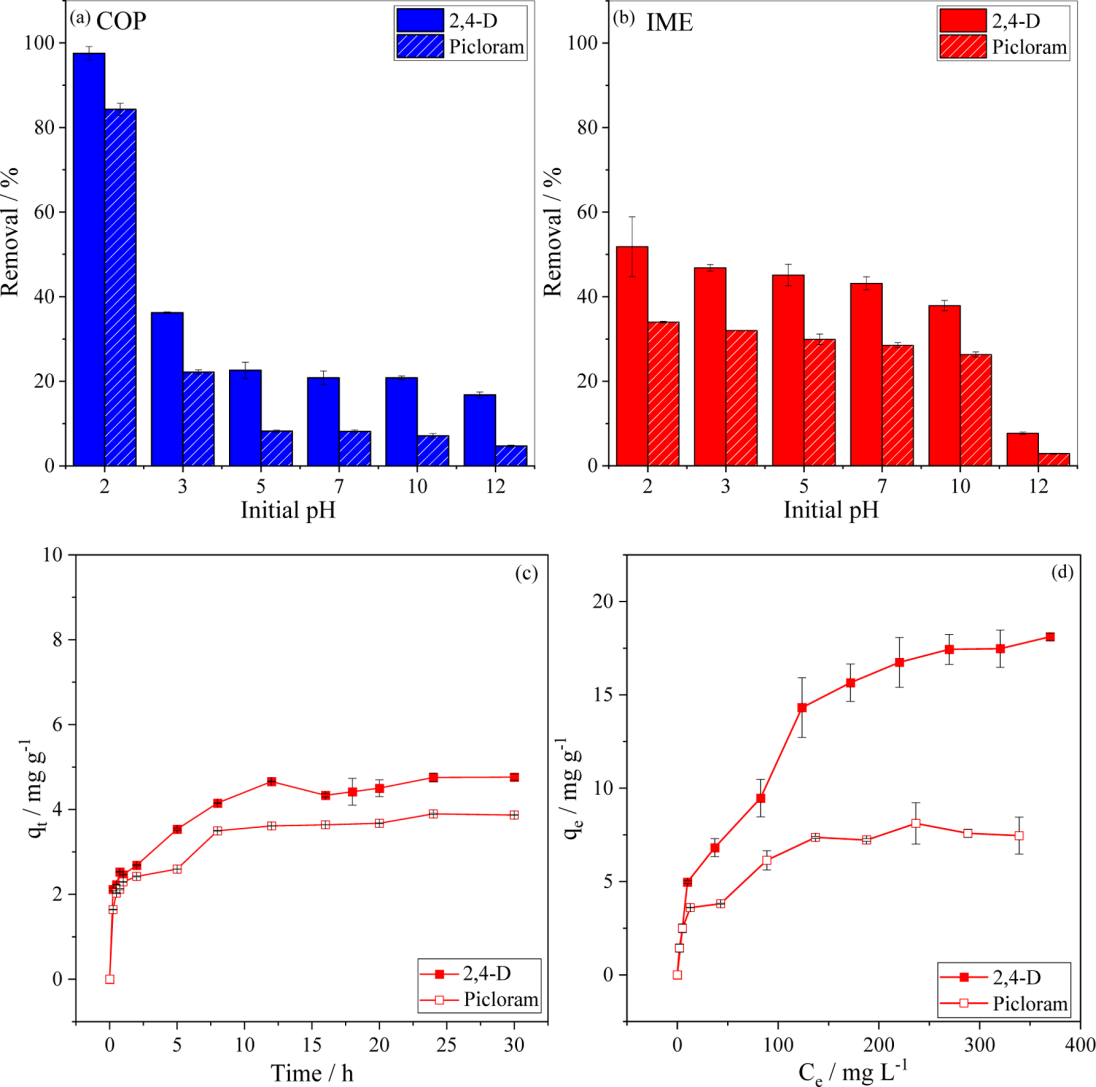
Effect of initial pH on 2,4-D and picloram adsorption on (a) COP and (b) IME. Effect of (c) contact time and (d) herbicide initial concentration on the adsorption on IME. Conditions: dose of 2.00 g L^–1^; 120 rpm; 25.0 °C; pH 5 (no adjustment) for 2,4-D and pH 4 (no adjustment) for Picloram; initial concentration of 20.0 mg L^–1^ of 2,4-D (or picloram) for (a,b,c); contact time of 24 h for (a,b,d).

The COP material showed a sharp decrease in 2,4-D removal efficiency as the pH increased from 2 to 3, dropping to approximately one-third of its initial value and remaining nearly constant at around 20% above pH 5. In contrast, IME maintained removal above 40% for 2,4-D within pH 2–8, with a pronounced decrease under strongly alkaline conditions (38% at pH 10 and 8% at pH 12). Notably, this steep reduction in removal percentage at high pH (10–12) was not observed for COP, consistent with their distinct pH_PZC_ values (4.2 for IME and 9.6 for COP, Table S4), which make IME more sensitive to surface charge changes under alkaline conditions. Further insight comes from ΔpH data (Table S11 and Figure S3). For IME, ΔpH changed only slightly between pH 3 and 5, indicating resistance to surface protonation, but showed marked variation between pH 6 and 11, reflecting higher deprotonation degree under alkaline conditions. COP displayed higher ΔpH values between pH 3 and 5, suggesting greater surface activity, with low ΔpH values in alkaline medium (pH >9). These findings align with the more intense effects of pH on adsorption observed for COP at low pH and for IME at high pH, underscoring the influence of protonation degree of the surface on herbicide removal. Similar trends were observed for picloram, although with slightly lower removal percentages.

The p*K*_a_ values of 2,4-D (2.7)^77^ and picloram (2.3)^78^ indicate that electrostatic attraction was not the dominant adsorption mechanism at pH 2.0. Moreover, if such interactions were predominant, increasing the ionic strength of the solution would have reduced the removal efficiency by shielding the attraction between herbicide molecules and adsorption sites. However, no significant variation was observed in the presence of higher NaCl concentrations (Figure S9), suggesting that other factors beyond electrostatic interactions are involved in the adsorption process. Instead, hydrogen bonding and complexation probably prevailed under acidic conditions, while at a higher pH, weaker interactions such as van der Waals forces or specific surface bonding mechanisms became more relevant (see Section 3.4 for an adsorption mechanism discussion). Differences in oxide crystallite size may also explain the observed behaviors. IME contains larger crystallites than COP, probably resulting in lower surface area per unit mass. The enhanced reactivity, confirmed by XPS analysis (Section 3.1.4), can also favor adsorption on COP under acidic conditions, where surface charge effects are stronger.

Results similar to those obtained for COP are widely reported in the literature, with pH 2 frequently identified as the most favorable condition for 2,4-D adsorption, regardless of the adsorbent used.^25,75,79,80^ To date, Fe–Mn biochar has not been reported for the adsorption of either 2,4-D or picloram; only monometallic biochars have been investigated for 2,4-D adsorption. A biochar modified with FeCl_3_ prior to pyrolysis and produced at 600 °C achieved quantitative removal (100%) across the pH range of 2–10 (20 mg L^–1^ 2,4-D; 2 g L^–1^),^27^ whereas an activated carbon impregnated with presynthesized Fe_2_O_3_ nanoparticles reached 98.12% removal at pH ∼2 (32 mg L^–1^ 2,4-D; 0.2 g L^–1^), followed by a sharp decrease in efficiency at higher pH values (24.40% at pH 6).^80^ A monometallic Mn-biochar produced at 400 °C via prepyrolysis modification with MnCl_2_ exhibited removals of 99% at pH 2 and 94% at pH 3, decreasing to 69% at pH 10 (20 mg L^–1^ of 2,4-D; 2 g L^–1^), confirming the strong dependence of adsorption performance on acidic conditions.^23^

The lower adsorption capacity of the bimetallic Fe–Mn biochar composites produced in this work compared with optimized monometallic systems already reported reflects a trade-off inherent to the modification strategy, as the simultaneous incorporation of both metals results in lower surface concentrations of highly active oxide phases and partial site shielding, particularly after postsynthesis washing steps implemented here to enhance metal stability and reduce leaching. Although the moderate removal efficiencies obtained with IME, its stability across the entire pH range demonstrates consistent performance, highlighting its potential as an adsorbent in systems subject to pH fluctuations.

It is also important to note that, although the highest removal efficiencies were observed under acidic conditions, such pH values are rarely encountered in real water matrices impacted by herbicides, which typically exhibit pH values between 6 and 9.^81^ Moreover, operation under strongly acidic conditions may increase the risk of metal leaching from the composite material, as evidenced by the leaching results discussed in subsequent sections. Therefore, adsorption performance under near-neutral conditions provides a more realistic and environmentally safe assessment of the material’s applicability in practical water treatment scenarios. Due to the lower variation in the adsorption capacity of the IME material within the studied pH range and considering the results obtained from the leaching tests (Section 3.5.1), subsequent adsorption experiments were carried out exclusively with IME.

#### 3.2.3 Dose Effect

Aiming at higher removal of the herbicides, experiments were carried out using different doses of IME (Figure S13). A significant increase in the percentage removal of herbicides (from 41.0% to 95.8% for 2,4-D and from 26.6% to 96.8% for picloram) was observed from 1 to 8 g L^–1^ of IME. This behavior can be attributed to the greater availability of active sites, which favors the adsorption process.^82^ Notably, increasing the IME dose from 2 to 4 g L^–1^ resulted in a substantial increase in herbicide removal, while further increments above 4 g L^–1^ produced only marginal gains. This indicates that beyond this point particle overlap occurs. The decrease in the amount adsorbed at equilibrium (mg g^–1^) as the dose increases is due to the increase in the availability of active sites.^83^

The adsorbent dose should be chosen to achieve an optimal balance between the removal performance and rational use of the material. For the purposes of this study and considering the environmental safety results (see Section 3.5), the experiments were conducted with a dose of 2 g L^–1^.

#### 3.2.4 Effect of Contact Time

The adsorption kinetic was evaluated to elucidate the effect of contact time on herbicide removal by the IME material. Figure 4c shows the amount adsorbed (*q*_e_) as a function of time for both herbicides, showing that adsorption equilibrium was reached after approximately 10 h for both picloram and 2,4-D. High adsorption is observed initially due to the large availability of active sites, followed by a gradual decrease in adsorption rate until reaching the steady state, as the remaining surface sites became progressively occupied.^75^

Faster equilibrium times have been reported for 2,4-D adsorption on different materials. For example, H_3_PO_4_^–^ modified biochar from spent coffee grounds (300 °C) reached equilibrium within 1 h, while HCl-assisted hydrothermal carbonization of *Vateria indica* fruit biomass (200 °C) achieved equilibrium in 2 h. In contrast, nitrogen-doped biochar from palm kernel shells pyrolyzed at 800 °C required up to 7 h.^75,79,84^ Although the equilibrium condition in this study was attained more slowly, most of the adsorption occurred within the first 2 h of contact.

Different kinetic models were applied to the adsorption curves of 2,4-D and picloram as functions of time (Figure S10). The Elovich model provided the best fit to the experimental data for both herbicides, as indicated by the highest *R* ^2^ values (≥0.978), lowest RSS (≤0.562 mg^2^ g^–2^), and lowest AIC (≤−32.73) (Table S12). The Elovich model describes adsorption kinetics by considering the influence of adsorbate diffusion to the adsorbent surface and the variation in adsorption energy as available sites become occupied.^85,86^

The fitting of experimental data to the intraparticle diffusion model was used to investigate the predominant mechanisms during the adsorption process (Figure S11). The results revealed the presence of multiple linear segments, each with a nonzero slope (C ≠ 0), indicating the coexistence of multiple mechanisms. This behavior suggests that surface adsorption, intraparticle diffusion, and film diffusion contribute to herbicide removal by IME. Marked differences between 2,4-D and picloram adsorption by IME were also observed. For 2,4-D, the diffusion coefficient associated with the first linear region (*K*_d1_) (Table S12) were 8-fold higher than those for picloram, indicating greater mobility and faster adsorption of 2,4-D in the intraparticle diffusion stage.

#### 3.2.5 Effect of Initial Concentration

At low initial concentrations, IME showed comparable adsorption capacities for 2,4-D and picloram (Figure 4d). However, at higher concentrations, 2,4-D exhibited a markedly greater adsorption capacity, reaching 18.11 mg g^–1^, while picloram stabilized at 8.11 mg g^–1^ at saturation. This behavior is consistent with the increasing concentration gradient that enhances diffusion of adsorbate molecules to surface and internal sites until the adsorbent becomes saturated.^87^ It is noteworthy that, in a previous study performed at similar pH values, a higher adsorption capacity for 2,4-D was reported when Fe was used (up to 30.94 mg g^–1^).^27^ This indicates that the presence of both Fe and Mn oxides in the composite may alter the accessibility of active sites through competitive effects, site blocking, or modifications in surface morphology and chemistry, ultimately limiting adsorption compared to single-oxide systems. In addition, the effective incorporation of oxides in the previous reported works must be considered, since herein the biomass was separated from the supernatant prior to pyrolysis, reducing the amount of metal available for oxide formation. However, most studies do not report the total Fe and Mn contents in the adsorbents, hindering a direct comparison of metal loadings.

Among the isotherm models evaluated to describe the adsorption of the herbicides by IME (Figure S12), the Sips model provided the best fit for both compounds, as indicated by the highest *R* ^2^ values (≥0.968), lowest RSS (≤8.286), and lowest AIC (≤5.26) values (Table S13). This model, which combines characteristics of the Langmuir and Freundlich models,^88^ is particularly suitable for heterogeneous systems, enabling accurate description of adsorption over a wide concentration range.

The greater *q*_e_ observed for 2,4-D compared to picloram may be attributed to the structural heterogeneity of IME, with a higher availability of preferential metal sites for 2,4-D. Additionally, the lower adsorption capacity observed for picloram may be related to its molecular structure and differences in charge distribution, which can limit its interaction with active sites on the IME surface and reduce the formation of specific interactions, such as complexation. This aspect of the mechanism will be discussed in more detail in Section 3.4.

### 3.3 Reuse

The reusability of IME was evaluated to assess its stability and economic feasibility, employing an alkaline solvent for desorption, commonly reported for Mn and/or Fe-modified materials.^23,27,89–91^ The results revealed a continuous decrease in removal efficiency for both herbicides over successive cycles (Figure S14a). For 2,4-D, removal decreased from 97.0% to 60.9% and 29.3% in the second and third cycles, respectively. Picloram followed a similar trend, with removal efficiencies of 82.4%, 45.1%, and 23.7%, respectively. Desorption efficiency also progressively decreased with each cycle (Figure S14b).

Notably, the high adsorption percentages observed in the reuse experiments (first cycle) result from the lower concentration used (10 mg L^–1^, half of that in previous experiments) and from the change in experimental configuration: due to the larger solution volumes, experiments were performed in Erlenmeyer flasks rather than vials. The Erlenmeyer geometry enhanced mixing and likely promoted particle dispersion or even breaking of particles, increasing the accessible surface area and improving mass transfer to the adsorbent. Consequently, the system exhibited more efficient contact between herbicide molecules and the adsorbent, resulting in a higher adsorption.

For picloram, desorption reached 100% in the first cycle, indicating complete removal of the adsorbed herbicide and, in principle, full regeneration of the active sites. However, the removal efficiency in the second cycle was markedly lower than that in the initial cycle. Despite the apparent regeneration of the adsorption sites, the adsorption–desorption process may have induced structural or surface modifications in the material, reducing the accessibility or affinity of active sites for subsequent picloram molecules. Furthermore, site passivation, blockage, or other physicochemical alterations may have contributed to the diminished uptake in successive cycles, also explaining similar result for 2,4-D.^92^

These results represent a limitation in terms of the reusability of the material and highlight the need for improving regeneration strategies or enhancing material stability for applications involving multiple cycles. In addition, treatment strategies emphasizing effective contaminant removal followed by safe handling, degradation, or disposal of the spent material may be more practical than extensive regeneration and reuse in continuous treatment systems.

### 3.4 Mechanism of 2,4-D and Picloram Adsorption on IME Surface

The differences in adsorption capacities of 2,4-D and picloram on IME highlight distinct interaction mechanisms between each herbicide and the adsorbent surface and can help explain the variations in adsorption performance observed between IME and COP.

Isotherms were obtained at pH values between 4 and 5, which are close to the point of zero charge (pH_PZC_ = 4.08) of IME. At this nearly neutral surface charge, both 2,4-D and picloram exist predominantly as anions, making electrostatic attractions with the surface unlikely. Consequently, the removal of these herbicides from solution is mainly governed by other interaction processes. Previous works on the removal of 2,4-D using Mn-modified biochar or Fe-modified biochar have shown that surface complexation involving carboxyl groups in 2,4-D and metal sites (Fe^27^ or Mn^23^) in the oxide play an important role in the process, being the main mechanism at high pH values.

To elucidate the underlying processes governing the removal of each herbicide, XPS spectra were analyzed before and after adsorption. XPS survey spectra after herbicide adsorption (Figure S15) revealed an increase in the atomic fraction of the oxygen, rising from 11.48% to 13.03% for 2,4-D and to 13.87% for picloram (Table S9). This increase indicates the incorporation of oxygen-containing groups from the adsorbed herbicide molecules. The increased Cl 2p and N 1s signals, particularly after picloram adsorption, further confirm the successful adsorption of this compound.

An increased Fe signal intensity on the surface after the adsorption of both herbicides was also observed. This points to the active role of Fe in the adsorption mechanism, confirming that Fe acts as a complexation center, forming metal–carboxylate surface complexes with herbicide functional groups, especially with 2,4-D.^27,93^ The pronounced reduction in carboxylic groups (O=C–O) in high-resolution spectra after adsorption supports this proposed mechanism.

In contrast, manganese exhibited a distinctive behavior. After 2,4-D adsorption, the Mn surface atomic percentage decreased sharply from 0.59% to 0.09%, and the Mn 2p_3/2_ peak shifted from 642.09 to 641.50 eV. This substantial reduction, beyond minor leaching detected in solution after adsorption (see Section 3.5.1), suggests that a significant portion of Mn may have been redistributed, encapsulated, undergone a change in oxidation state, or directly participated in surface interactions with 2,4-D, as evidenced by the binding energy shift. The observed shift indicates modifications in the oxidation state or electronic environment of Mn, consistent with its involvement in redox or surface complexation processes. The atomic percentage of Mn was the same before and after adsorption of picloram (0.57%), and the peak position remained essentially unchanged (642.10 eV), implying no significant mobilization or chemical alteration of Mn and, therefore, a negligible role of Mn in picloram adsorption.

High-resolution spectra (Figures S16 and S17 and Table S10) provide further mechanistic insight. For 2,4-D, a marked decrease in C–C/C=C (from 75.3% to 71.5%) accompanied by a substantial increase in C–O–C (from 8.8% to 19.0%, with a peak shift from 286.5 to 286.1 eV) indicates the participation of oxygenated groups in 2,4-D and their interaction with surface metals on IME. The decrease in O=C–O fraction (from 10.97% to 9.53%) supports the formation of metal–carboxylate complexes, primarily involving Fe^3+^,^94,95^ as corroborated by the increase in Fe^3+^ (2p_3/2_) from 20.07% to 23.52% and its peak shift from 713.2 to 712.6 eV. In addition, the rise in the metal–O fraction of O 1s from 1.76% to 5.18% reinforces the role of surface metallic sites in mediating 2,4-D binding.

For picloram, adsorption was marked by an increase in C–OH (from 4.92% to 6.95%) and O=C–O (from 10.97% to 12.84%), indicating polar interactions and some degree of complexation. However, C–O–C remained unchanged (7.14%), Fe^3+^ (2p_3/2_) remained almost constant (18.02%) without a peak shift, and Mn remained constant both in atomic percentage and binding energy, indicating a minor role for these metals.

Under the pH conditions evaluated, classical hydrogen bonding is unlikely to be dominant, as both the adsorbent and herbicides are mostly deprotonated. Nonetheless, weak hydrogen bonds involving residual surface –OH or water cannot be fully excluded.

Physical mechanisms such as π–π stacking between the aromatic rings of the herbicides and the graphitic domains of the biochar, pore filling, and van der Waals interactions can also contribute to the retention of herbicides, especially 2,4-D.^23,96^ The more rigid and symmetrical structure of picloram, lower density of accessible functional groups, and possible steric hindrance restrict its effective interaction with IME active sites.

In summary, XPS results confirm that the adsorption of 2,4-D and picloram by IME occurs via multiple mechanisms: surface complexation (mainly with Fe^3+^), interactions with oxygenated groups, π–π stacking, pore filling, and van der Waals forces.

### 3.5 Environmental Safety of the Adsorbents

#### 3.5.1 Fe and Mn Leaching

It is important to emphasize the need for comprehensive environmental safety studies of adsorbents, including assessments of secondary contaminant generation, to better understand the environmental stability and practical implications of Fe–Mn biochar composites in real-world applications. Leaching studies of Mn and Fe were performed to assess the stability of the impregnated metals in the adsorbents. Both COP and IME were evaluated at different pH values during the adsorption of 2,4-D (Figure 5a,b) with the aim to assess their environmental safety for potential applications and to elucidate how the modification process influences metal retention within the composites.

**Figure 5:**
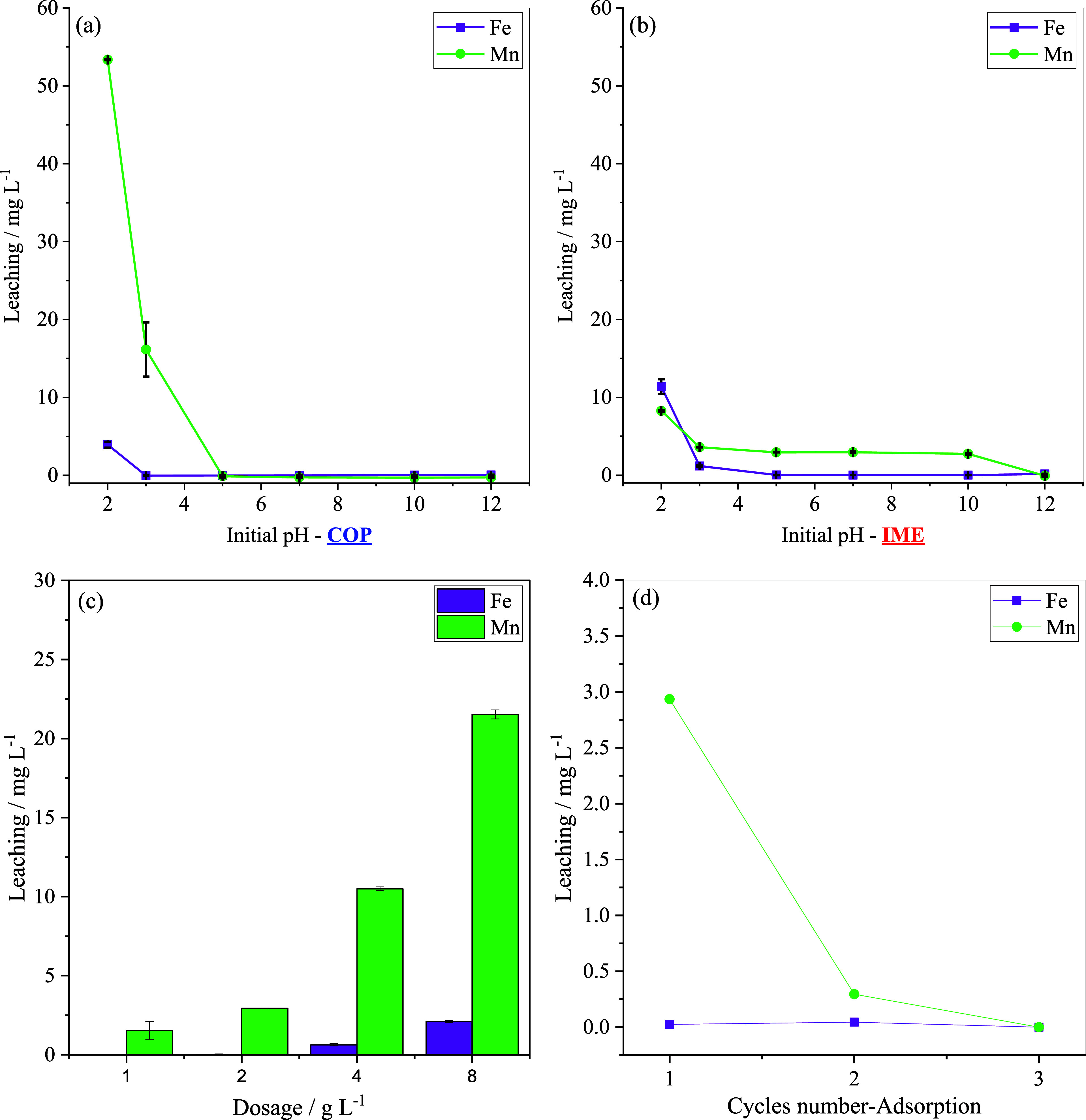
Fe and Mn leaching from COP (a) and IME (b) at different pH values, from IME at increasing adsorbent doses (c), and during successive reuse cycles in IME adsorption (d). Experimental conditions: 120 rpm, 25 °C, 20.0 mg L^–1^ 2,4-D, 24 h contact time, adsorbent dose of 2.00 g L^–1^ for (a–d); pH 5 for (c,d). Data obtained from FAAS analysis.

Under acidic conditions (pH 2), IME exhibited high Fe leaching (11.38 mg L^–1^), while COP showed a considerably lower value (3.93 mg L^–1^). At pH 3, Fe leaching in IME drastically decreased to 1.18 mg L^–1^, while COP maintained negligible values (below the LOD of FAAS), suggesting greater stability of iron in the COP matrix under slightly acidic conditions.

For Mn, COP exhibited markedly higher leaching at pH 2 (53.36 mg L^–1^), whereas IME exhibited substantially less leaching (8.27 mg L^–1^). As pH increased, Mn leaching from COP declined sharply up to pH 5, while IME maintained a relatively constant Mn release (2.70–3.50 mg L^–1^) in the range from 3 to 10, reflecting stability of manganese under this pH range.

Hydrolysis of the released metal ions at higher pH values should also be considered in the previously reported results. Therefore, to validate these findings, the elemental composition of the adsorbents after the leaching experiments was determined by ICP-OES (Table S8). In COP, Mn changed from 128.3 g kg^–1^ (initial condition, before leaching) to 122.7 g kg^–1^ at pH 2 and to 116.6 g kg^–1^ at pH 7, indicating moderate Mn mobilization. At pH 12, however, Mn was largely retained (125.5 g kg^–1^), suggesting reprecipitation or stabilization of Mn species on the adsorbent. A similar behavior was observed for Fe: concentrations declined from 141.4 mg kg^–1^ (initial) to 122.9 mg kg^–1^ at pH 2 and 118.3 mg kg^–1^ at pH 7, but recovered to near-initial levels at pH 12 (139.3 mg kg^–1^). IME exhibited lower initial Mn (20.2 g kg^–1^) and Fe (53.7 mg kg^–1^) amount than COP. At pH 2, Mn dropped to 12.4 g kg^–1^ and Fe dropped to 35.8 mg kg^–1^, representing higher relative losses under acidic conditions compared with neutral or alkaline media. At pH 7, Mn and Fe contents increased (16.1 g kg^–1^ and 40.4 mg kg^–1^, respectively), while at pH 12, they approached the original values (18.7 g kg^–1^ and 51.4 mg kg^–1^).

These results confirm that acidic and near-neutral conditions promote Mn and Fe release from COP and IME, whereas alkaline conditions favor their retention in the solid matrix. The observed trends are largely consistent with the extent of metal leaching after adsorption, where Fe and Mn concentrations in the leachate at pH 12 were lower than those at pH 2. However, they diverge at pH 7, where the leachate concentrations of both metals were comparable to those at pH 12. This discrepancy suggests that under neutral conditions, Mn and Fe likely underwent precipitation but remained weakly anchored to the adsorbent surface, thereby facilitating their subsequent removal during the washing step.

Overall, ICP-OES analysis showed that Mn was more prone to leaching than Fe in both composites, especially under acidic conditions. COP, with higher metal loadings, exhibited greater absolute leaching but also stronger stabilization at alkaline medium, although under these conditions its herbicide adsorption capacity is reduced. IME, in contrast, incorporated lower amounts of metals and, despite proportionally larger relative losses, maintained 2,4-D removal efficiencies of >40% within the evaluated pH range (Section 3.2.2). This difference suggests that the excessive Mn release from COP under acidic conditions not only compromises its environmental safety but also makes its performance strongly pH-dependent, whereas IME demonstrated more consistent adsorption behavior despite its lower metal content.

Beyond pH effects, Fe and Mn leaching from IME was also evaluated as a function of the adsorbent dose (Figure 5c). As expected, metal release increased with increasing dose: Fe remained negligible at low concentrations (<LOQ at 1 g L^–1^ and 0.025 mg L^–1^ at 2 g L^–1^) but rose to 0.63 mg L^–1^ at 4 g L^–1^ and 2.10 mg L^–1^ at 8 g L^–1^. Mn exhibited more pronounced leaching, increasing from 1.54 mg L^–1^ (1 g L^–1^) to 21.52 mg L^–1^ (8 g L^–1^).

On the other hand, leaching decreased with successive reuse (Figure 5d): in the first cycle, Fe and Mn releases were 0.025 and 2.94 mg L^–1^, respectively; in the second cycle, Fe slightly increased (0.045 mg L^–1^), while Mn decreased sharply (0.30 mg L^–1^); finally, in the third cycle, both metals were not detected. The higher Mn release observed in the first adsorption cycle can be attributed to the dissolution of weakly bound or more labile Mn species located on the external surface of the composite, which are more susceptible to mobilization upon initial contact with the leaching medium.

The progressive reduction in Mn leaching was accompanied by reduced herbicide adsorption efficiency of IME (Section 3.3), likely reflecting site saturation by nondesorbed molecules or a reduced availability of surface-associated metal species. In contrast, desorption assays conducted under alkaline conditions showed no detectable Fe or Mn leaching, probably due to NaOH-induced reprecipitation, which immobilized the metals on the composite surface.

#### 3.5.2 Phytotoxicity Assays

In the previous section, the release of secondary contaminants from IME during 2,4-D adsorption was discussed, with both Fe and Mn released at pH 5 (0.025 mg L^–1^ and 2.94 mg L^–1^, respectively, after adsorption assays) (Figure 5b). In Brazil, the regulatory limits for drinking water and for the irrigation of raw-consumed vegetables are 0.3 mg L^–1^ for Fe and 0.1 mg L^–1^ for Mn.^97,98^ Under these conditions, Fe remained within the limits while Mn exceeded the allowed value. To assess the biological relevance of these results, phytotoxicity tests were conducted, enabling a practical evaluation of whether residual herbicides or metal leaching compromises the safety of reusing treated water in agriculture.

Phytotoxicity assays with *C. sativus* seedlings revealed pronounced differences in morphological development among treatments (Figure 6), with the evaluated parameters and statistical details provided in Figure S18. All treatments achieved 100% germination, indicating that neither 2,4-D nor Mn released from IME interfered with the germination process (day 3). However, postgermination growth (day 8) was significantly impaired in seedlings exposed to 2,4-D (T2), with marked reductions in shoot height, stem diameter, root length, and biomass, consistent with the well-documented phytotoxic effects of 2,4-D, which disrupts hormonal regulation and inhibits cell elongation.^6,7^

**Figure 6:**
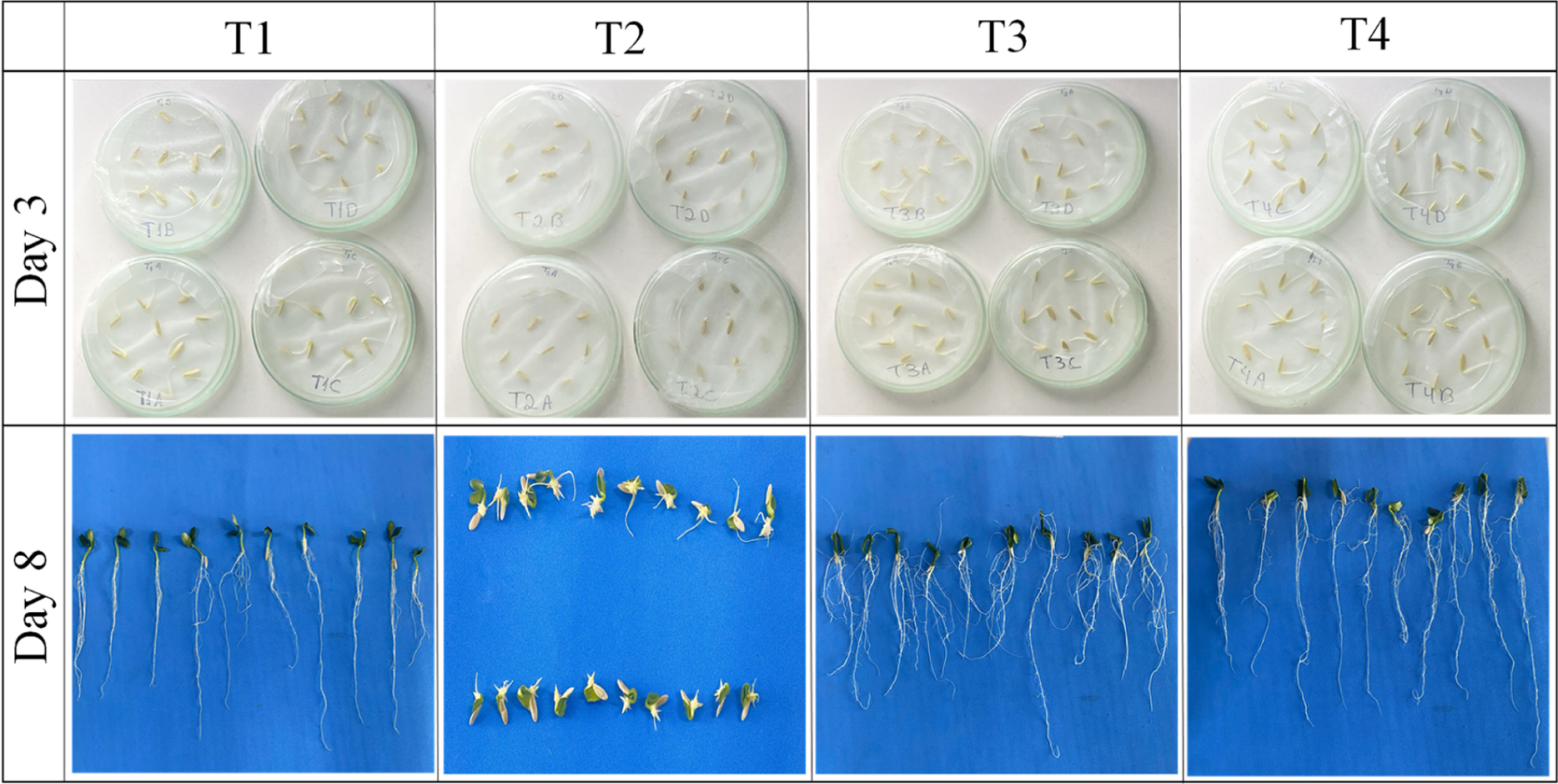
Phytotoxicity assays in cucumber seeds under different water treatments: deionized water (T1), 5 mg L^–1^ 2,4-D solution (T2), 5 mg L^–1^ 2,4-D solution after treatment with IME (T3), and solution containing only water after contact with IME in the absence of 2,4-D (T4).

In contrast, seedlings irrigated with 2,4-D solutions previously remediated by IME (T3) displayed morphological parameters statistically indistinguishable from the control (T1) and from the Mn-only treatment (T4), demonstrating effective mitigation of herbicide toxicity. The Mn released during adsorption (2.94 mg L^–1^) did not cause perceptible phytotoxic effects, likely due to its role as an essential micronutrient for *C. sativus*. Treatment T4 further confirmed that Mn leaching from IME alone did not impair the seedling development.

Overall, these results reinforce the high phytotoxicity of 2,4-D and demonstrate the efficacy and environmental safety of IME for remediating 2,4-D-contaminated water, combining efficient decontamination with minimal ecological risk. Nevertheless, in the context of water reuse for irrigation, it is crucial to ensure efficient herbicide removal to prevent residual toxicity.

Overall, IME demonstrated the effective removal of 2,4-D under the investigated conditions; however, Mn leaching exceeded guideline values for drinking water and irrigation, which limits its applicability for potable water treatment and unrestricted agricultural reuse. Notably, phytotoxicity assays showed no detectable adverse effects under the tested conditions, suggesting that Mn release is more relevant from a regulatory standpoint than immediate biological toxicity. Nevertheless, further studies are necessary to assess long-term implications, particularly regarding Mn accumulation and potential bioaccumulation in plants after repeated or prolonged exposure.

## 4 Conclusion

This study presents a comprehensive evaluation of the removal of herbicides 2,4-D and picloram using Fe–Mn oxide-modified biochars derived from sugarcane bagasse, emphasizing the influence of the modification strategy, adsorption mechanisms, and environmental safety. Two synthesis routes—immersion and coprecipitation—yielded composites with distinct surface chemistries, crystallinities, and adsorption behaviors. The IME material, characterized by a more amorphous and hydrophobic surface with larger oxide crystallites, exhibited consistent but moderate adsorption across a broad pH range, with enhanced 2,4-D removal primarily governed by surface complexation with Fe^3+^ sites. In contrast, COP displayed a higher crystallinity and greater surface oxygenation; however, its adsorption performance was more strongly pH-dependent and was accompanied by increased Mn leaching.

Mechanistic insights from XPS revealed that 2,4-D removal occurred primarily via the formation of Fe–carboxylate complexes, whereas picloram adsorption was governed by predominantly polar interactions involving surface oxygenated groups, with additional contributions from nonspecific interactions such as π–π and van der Waals forces, with limited participation of surface metals. The simultaneous incorporation of Fe and Mn oxides modified site accessibility and reactivity, constraining maximum adsorption capacities relative to those of single-metal systems reported in the literature.

Environmental assessments confirmed the superior stability of IME, with minimal Fe and moderate Mn leaching under environmentally relevant conditions. Mn release exceeded potability thresholds, underscoring the need for operational optimization or post-treatment when water reuse applications. Importantly, phytotoxicity assays demonstrated that IME-remediated water effectively mitigated 2,4-D toxicity in *C. sativus* seedlings, with Mn leaching exerting no detectable phytotoxic effects.

Collectively, these findings establish Fe–Mn biochar composites, particularly those produced by immersion, a simpler method, as promising adsorbents for the remediation of herbicide-contaminated waters. By elucidating the structure–function–safety relationships of Fe–Mn-based biochars, this work advances the rational design of high-performance and environmentally safer adsorbents for sustainable water treatment.

## Supplementary Material



## Data Availability

The data supporting the findings of this study are available within the article and the Supporting Information. Additional raw data are available from the corresponding authors upon reasonable request.

## Notes

Schematic diagram of materials production; conductometric titration curves and pH_PZC_ determination; EDS mapping and elemental composition; TGA profiles; preliminary adsorption tests and ionic strength effects; adsorption kinetics and isotherm modeling; adsorbent dose and regeneration cycles; XPS survey and high-resolution deconvoluted spectra; phytotoxicity assays; physicochemical properties of herbicides; kinetic and isotherm equations; FTIR band assignments; yield, surface acidity/basicity and crystallographic parameters; ICP-OES metal leaching data; XPS atomic percentages and functional group distribution; pH_PZC_ and ΔpH values; and estimated kinetic and isotherm parameters (PDF)
