# Supplementary material for: Fe–Mn Biochar Composites from Sugarcane Bagasse for Herbicides Removal: Structure, Mechanisms, and Safety Relationships toward Sustainable Water Treatment
Source: ACS Omega. 2026 Jan 29;11(5):8704–21. doi: 10.1021/acsomega.5c12317 (PMC12903173; doi:10.1021/acsomega.5c12317)
Supplement: Supplementary file 1 [file ao5c12317_si_001.pdf]

Supporting information

Fe-Mn–biochar composites from sugarcane bagasse for herbicides removal: structure, mechanisms, and safety relationships toward sustainable water treatment

Thamiris Ferreira Souza[a,*], Lucas Ferreira Fernandes[a], Laura Maria da Silva Batista[a], Carlos Henrique Milagres Ribeiro[b], Fábio Rodrigo Piovezani Rocha[c], Rafael Pio[b], Guilherme Max Dias Ferreira[a,**]

[a]*Group of Materials, Interfaces, and Solutions (MatIS), Department of Chemistry, Federal University of Lavras, Campus Universitário, PO Box 3037, Lavras, MG, Brazil.*

[b]*Department of Agriculture, Federal University of Lavras, Campus Universitário, PO Box 3037, Lavras, MG, Brazil.*

[c]*Center for Nuclear Energy in Agriculture (CENA), University of São Paulo, Av. Centenário, 303, 13416-000 Piracicaba, SP, Brazil.*

Corresponding authors:
**E-mail address: thamiris.f.souza@hotmail.com; Orcid: https://orcid.org/0000-0001-6775-6767*
***E-mail address: guilherme.ferreira@ufla.br; Orcid: https://orcid.org/0000-0002-4762-2777*

S1. Supplementary Figures

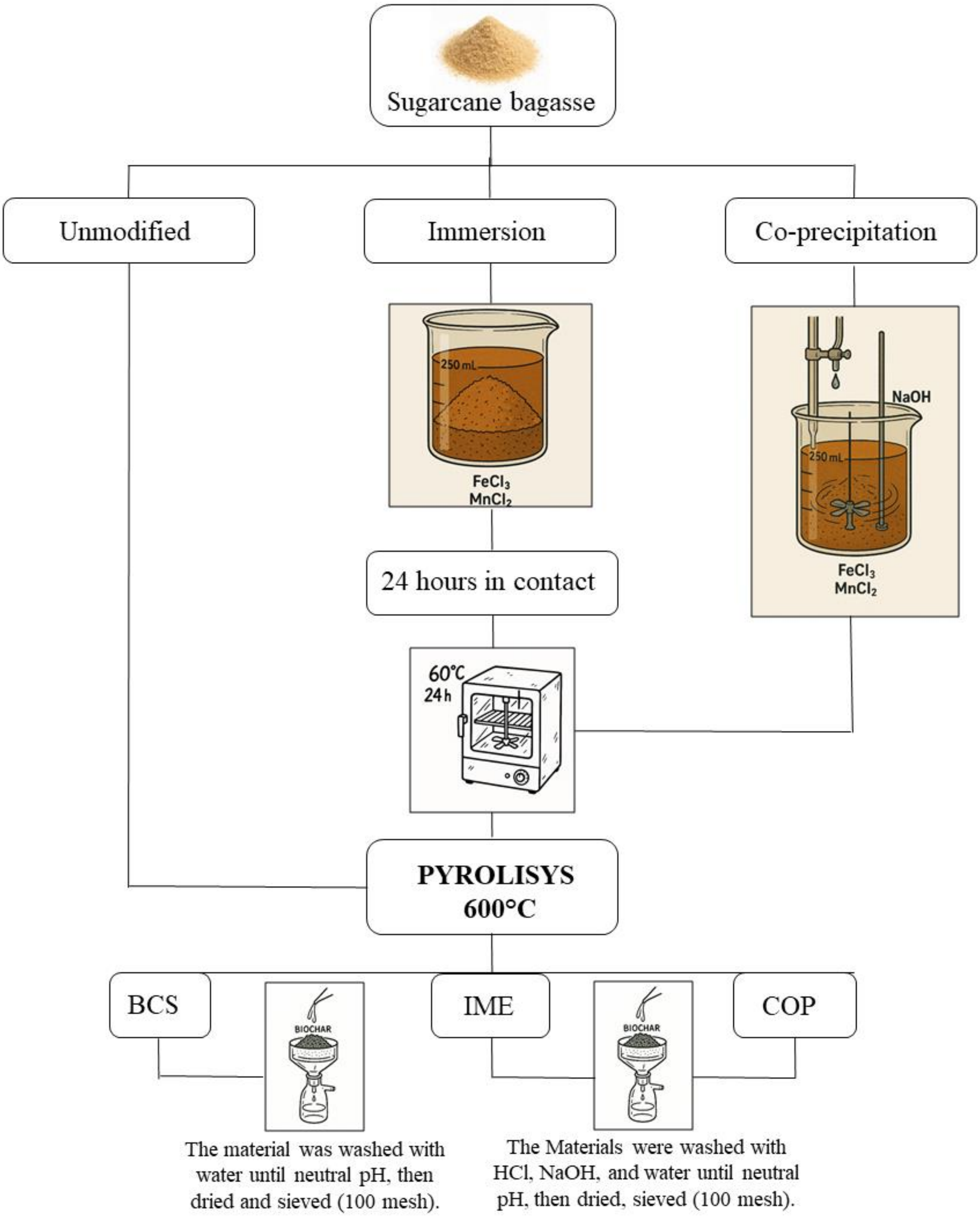

Figure S1. Schematic diagram of materials production.

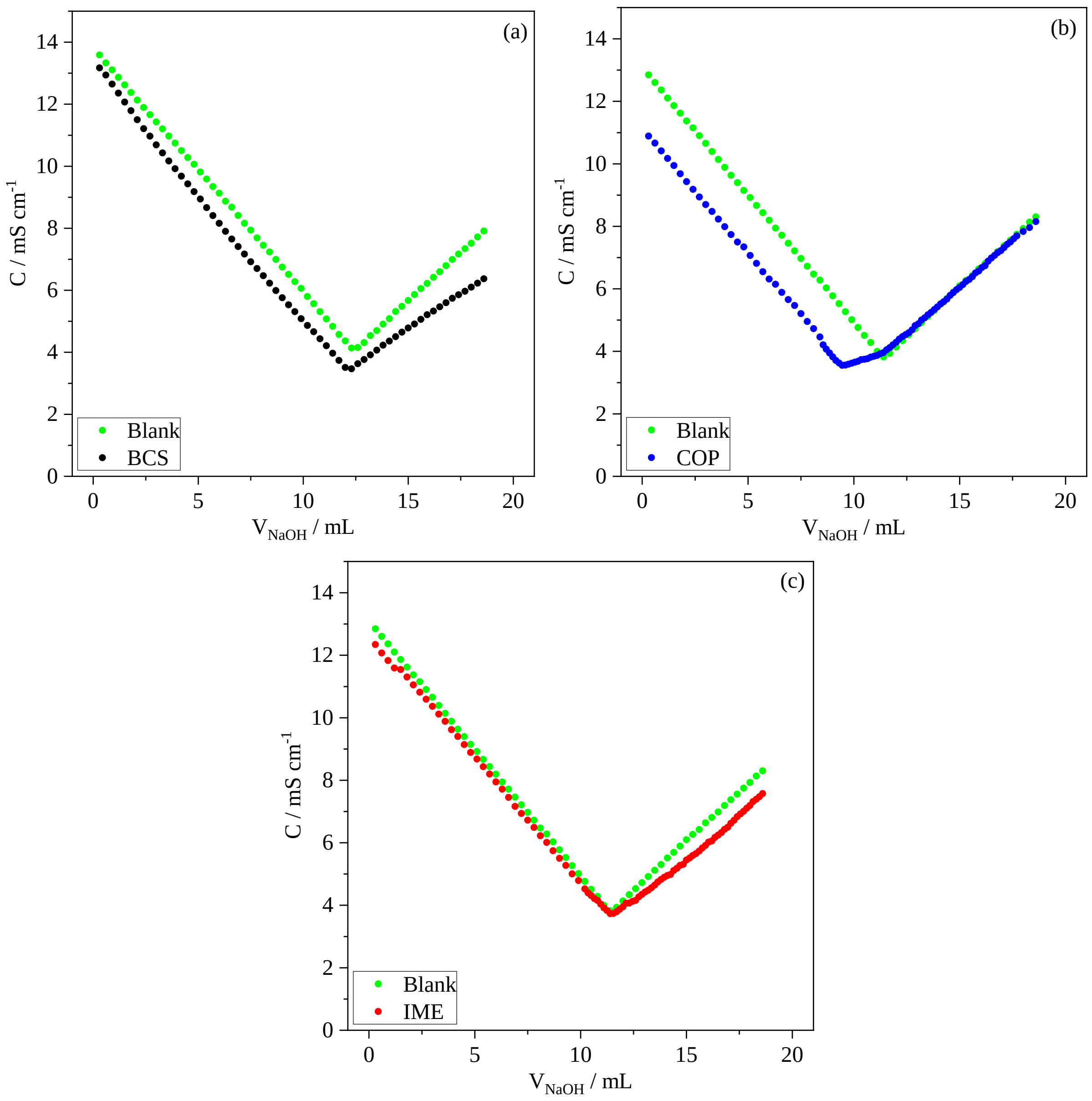

Figure S2. Conductometric curves for the titration of 70.00 mL of 0.035 mol L^{-1} HCl by 0.20 mol L^{-1} NaOH, in the absence (Blank) and in the presence of 0.1500 g of each biochar: (a) BCS, (b) COP, and (c) IME, at 25.0 °C .

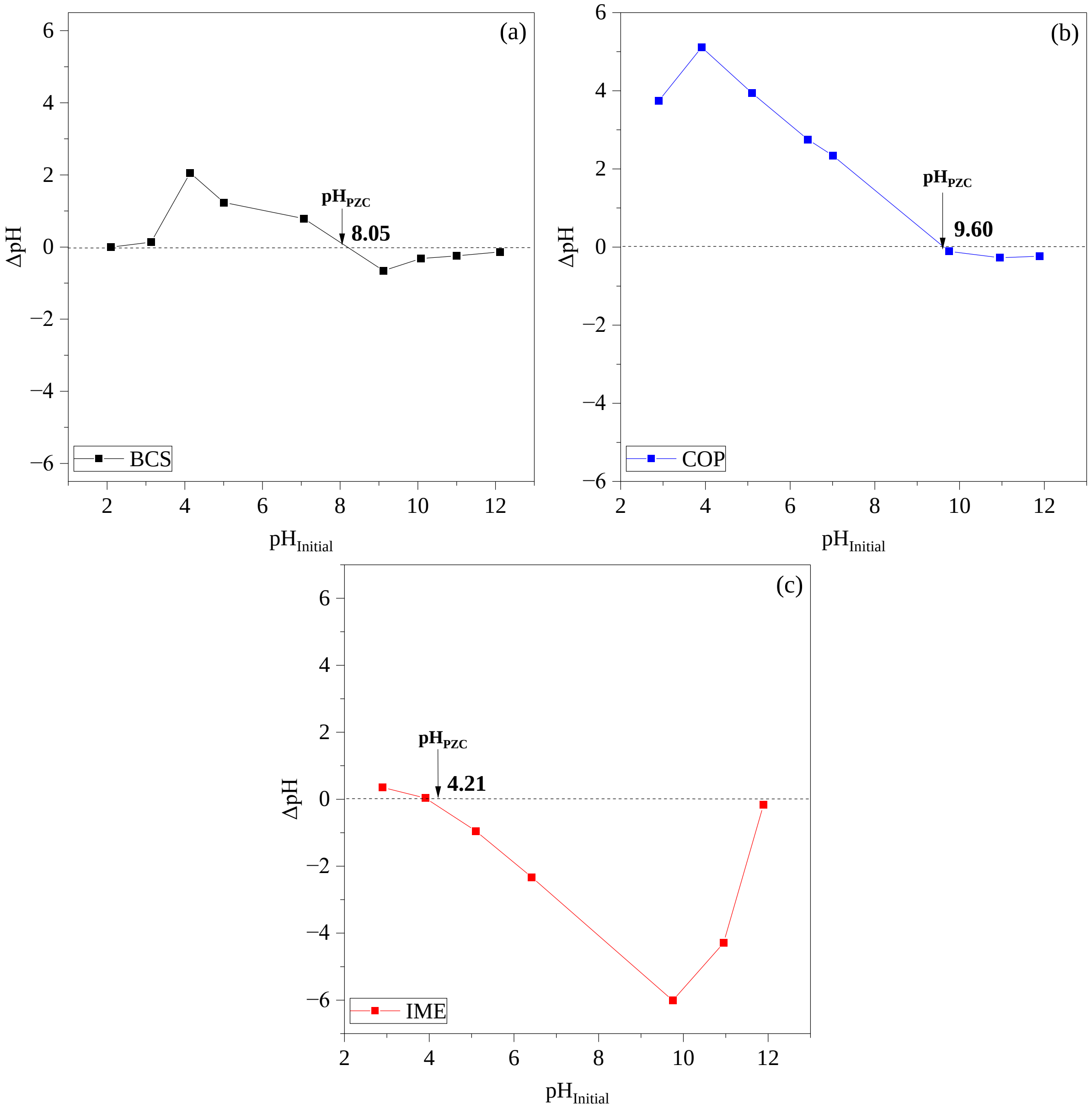

Figure S3. ΔpH versus pHi curves for the determination of the point of zero charge of each biochar: (a) BCS, (b) COP, and (c) IME.

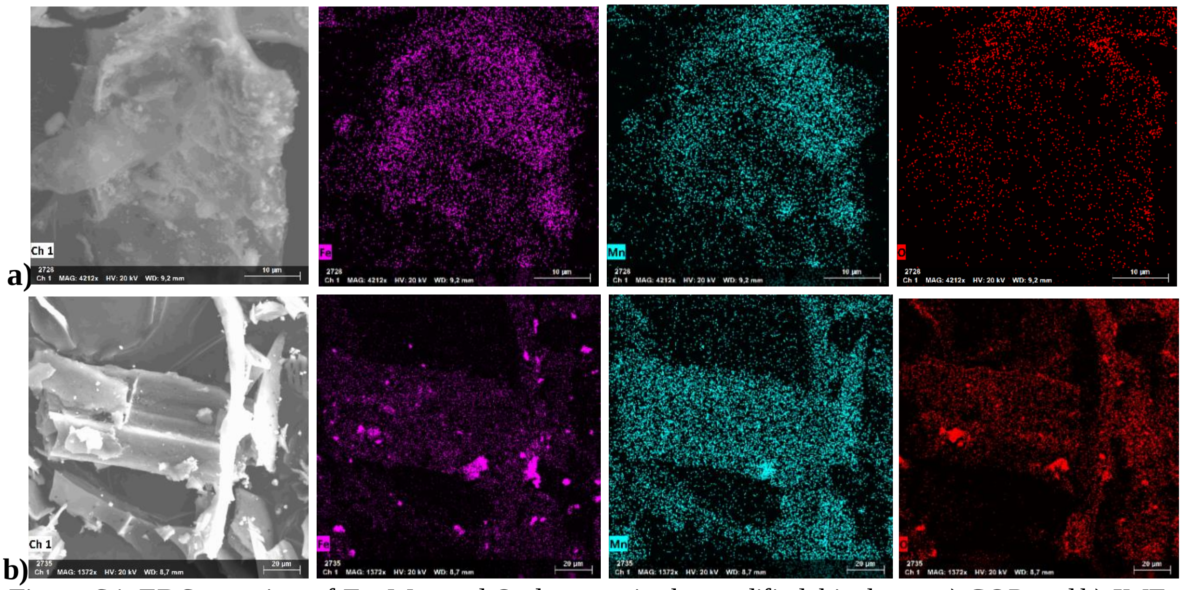

Figure S4. EDS mapping of Fe, Mn, and O elements in the modified biochars: a) COP and b) IME.

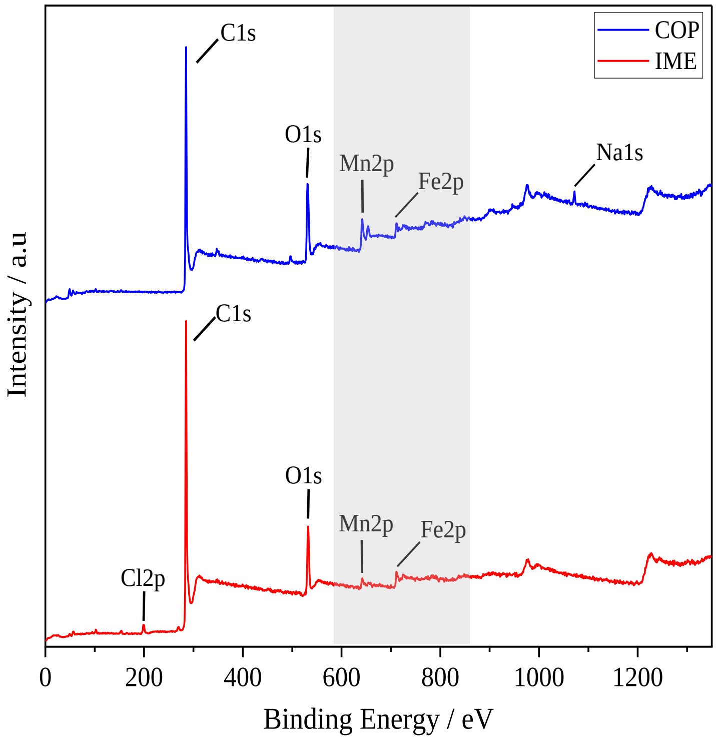

Figure S5. XPS survey spectra of IME and COP material.

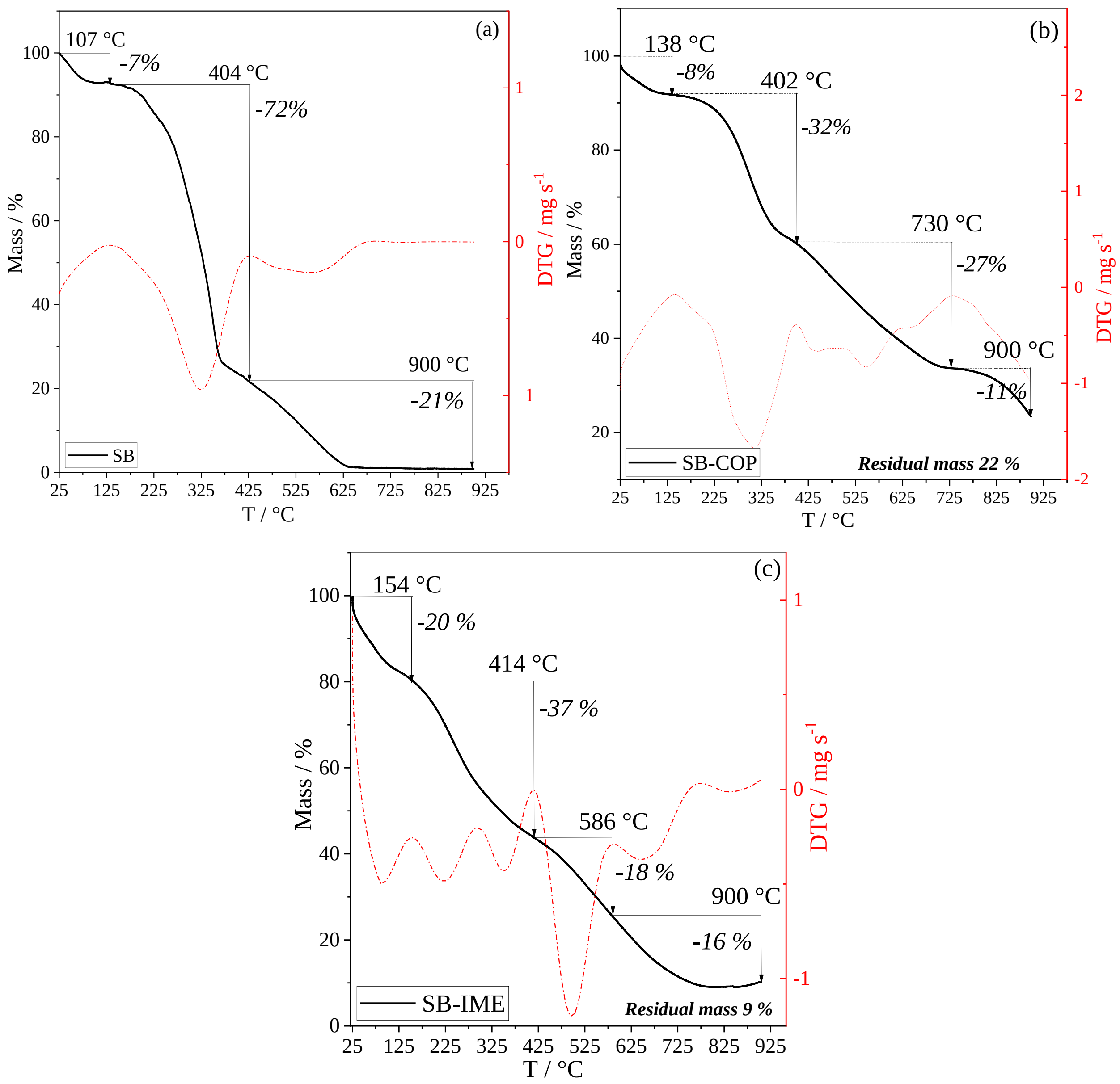

Figure S6. Stages of mass loss for (a) SB (b) SB-COP and (c) SB-IME. Temperatures on the arrows indicate the point of start or end of the degradation stage.

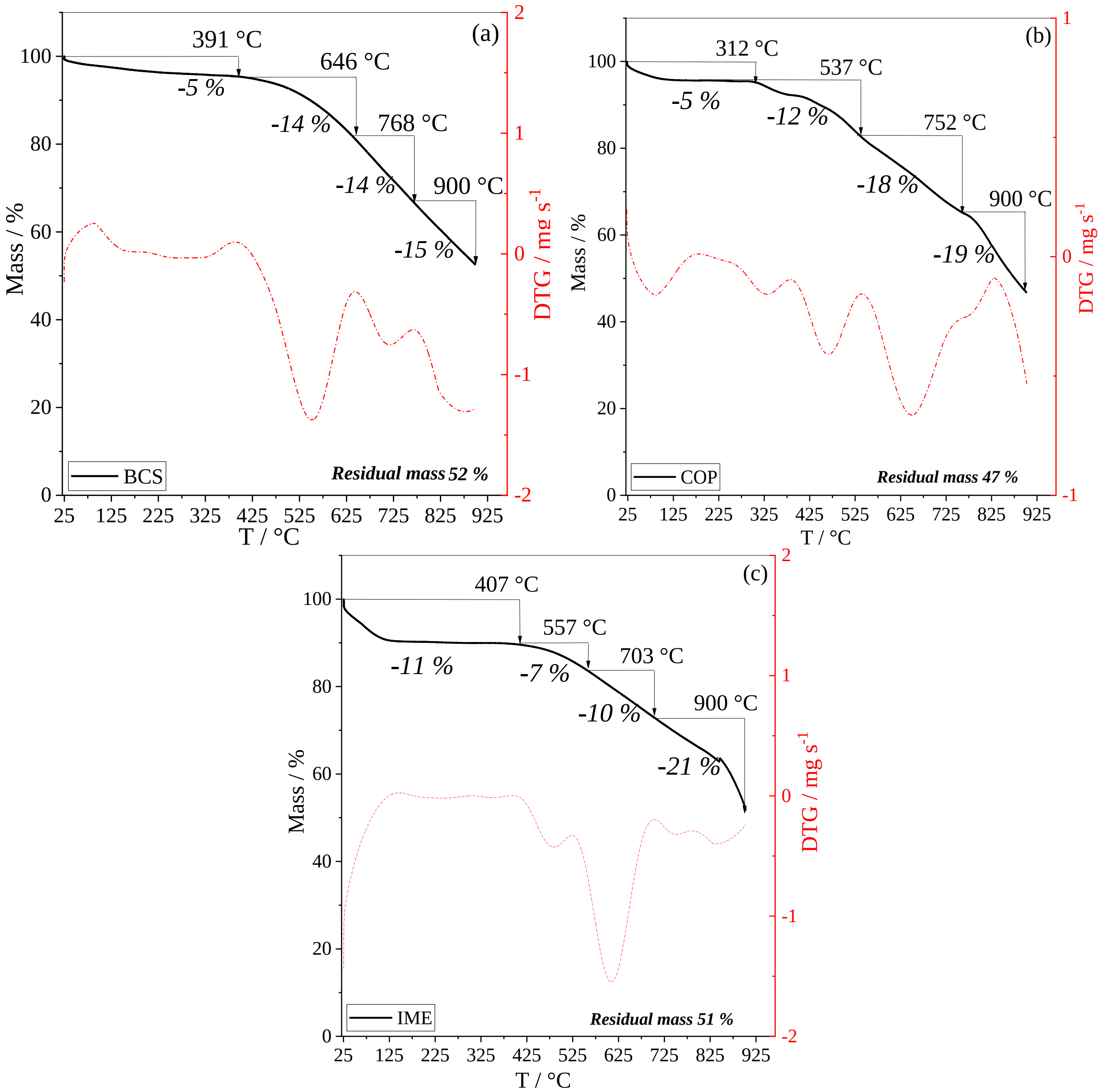

Figure S7. Stages of mass loss for (a) BCS, (b) COP and (c) IME. Temperatures on the arrows indicate the point of start or end of the degradation stage.

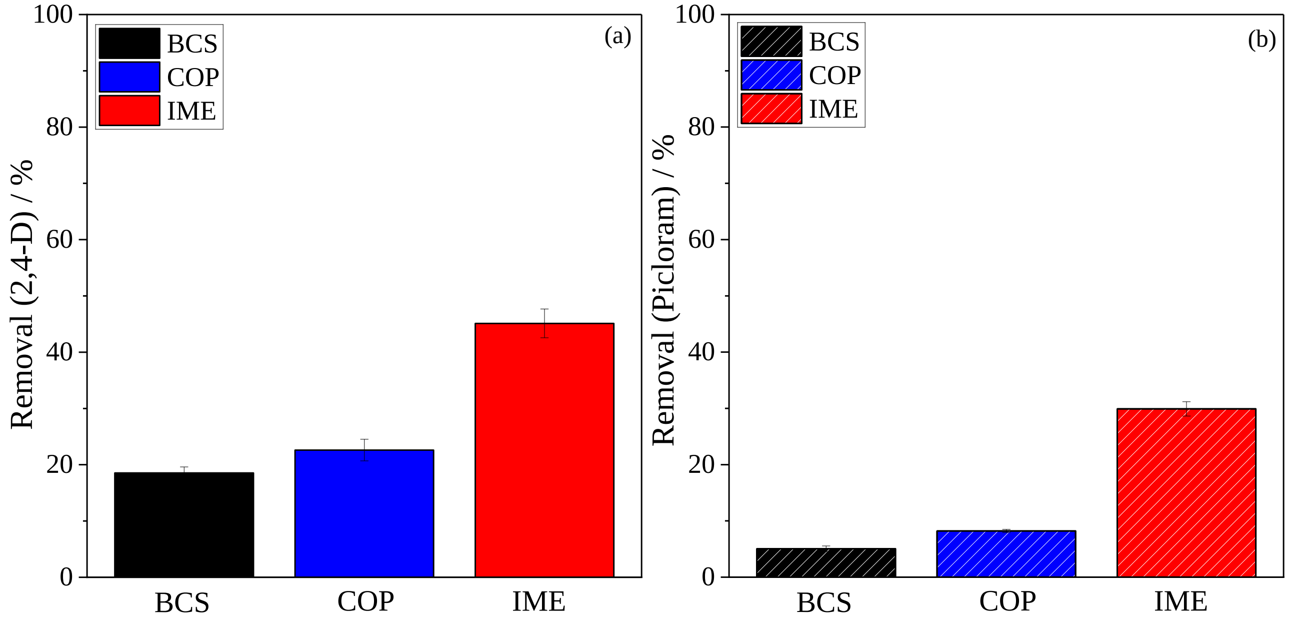

Figure S8. Adsorption preliminary test for 2,4-D (a) and Picloram (b). Conditions: 20.0 mg L^{-1} of 2,4-D or 4-Picloram; 2.00 g L^{-1} of adsorbent; pH around 5 (no adjustment) for 2,4-D and pH around 4 (no adjustment) for Picloram; 120 rpm; and 25.0 °C.

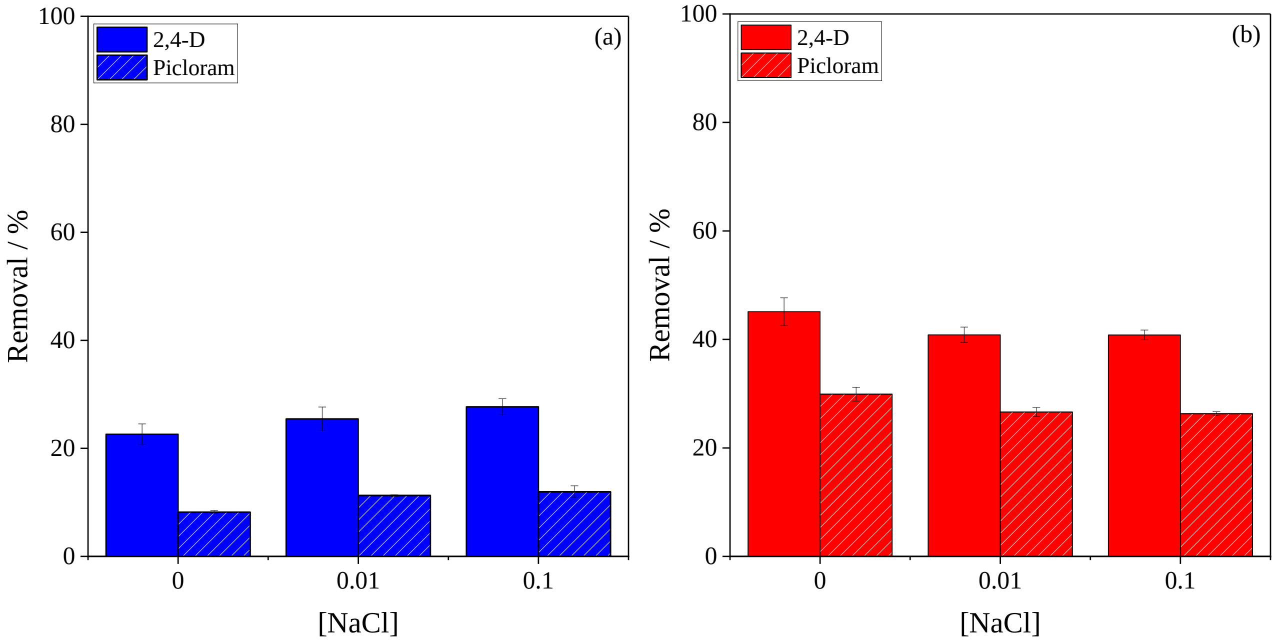

Figure S9. Effect of ionic strength on the adsorption of 2,4-D (a) and Picloram (b). Conditions: 20.0 mg L^{-1} of 2,4-D or 4-Picloram; 2.00 g L^{-1} of adsorbent; pH around 5 (no adjustment) for 2,4-D and pH around 4 (no adjustment) for Picloram; 120 rpm; and 25.0 °C.

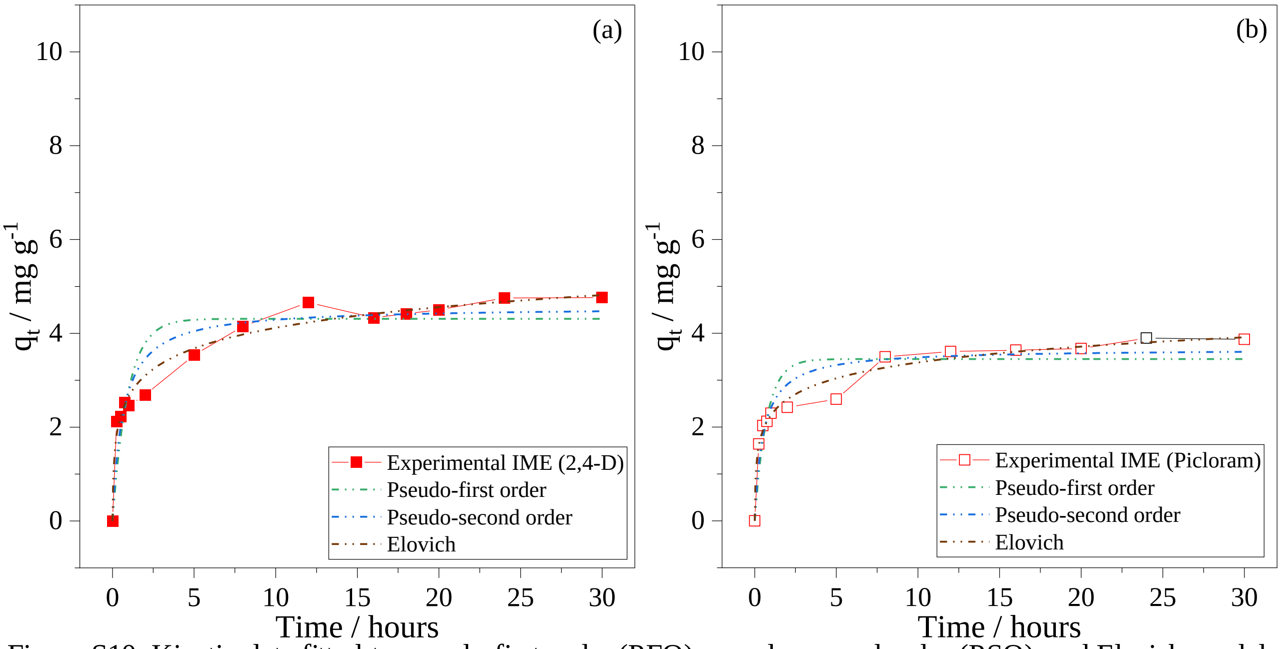

Figure S10. Kinetic data fitted to pseudo-first order (PFO), pseudo-second order (PSO), and Elovich models for 2,4-D(a) and Picloram(b) adsorption on IME. Conditions: 20.0 mg L^{-1} of 2,4-D or Picloram; 2.00 g L^{-1} of BC; range time: 0-30 h; pH around 5 (no adjustment) for 2,4-D and pH around 4 (no adjustment) for Picloram; 120 rpm; and 25.0 °C.

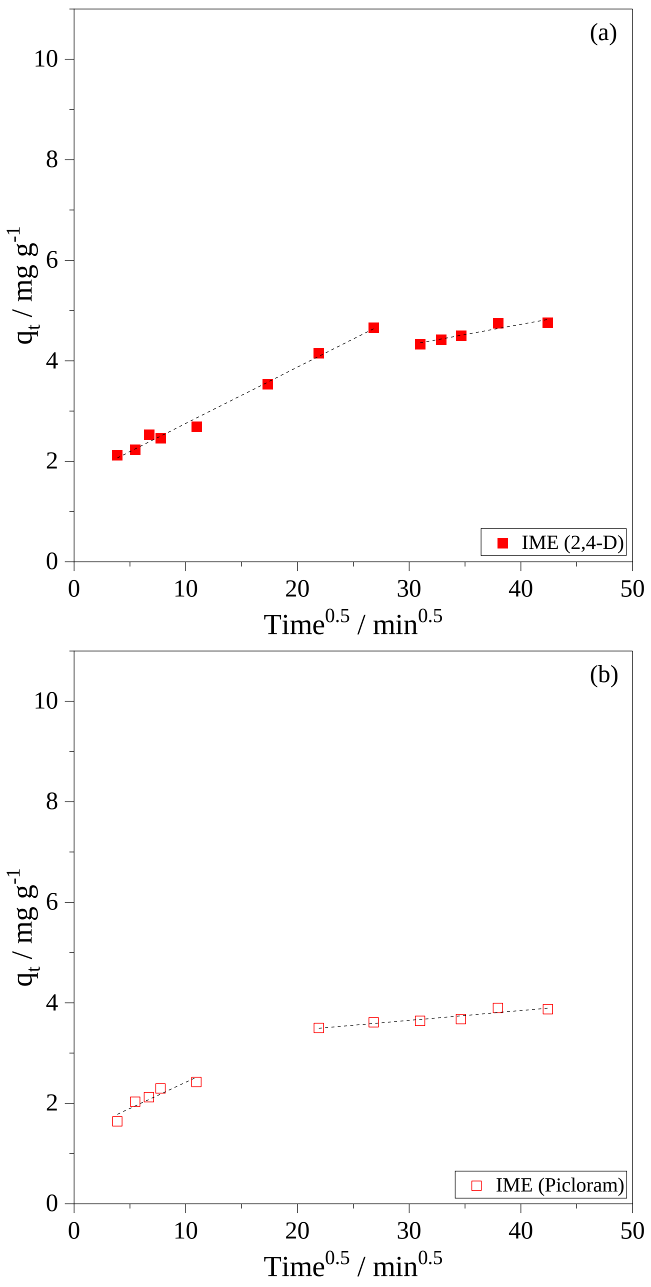

Figure S11. Kinetic data fitted to the intraparticle diffusion model for 2,4-D(a) and Picloram(b) adsorption on IME. Conditions: 20.0 mg L^{-1} of 2,4-D or Picloram; 2.00 g L^{-1} of BC; range time: 0-30 h; pH around 5 (no adjustment) for 2,4-D and pH around 4 (no adjustment) for Picloram; 120 rpm; and 25.0 °C.

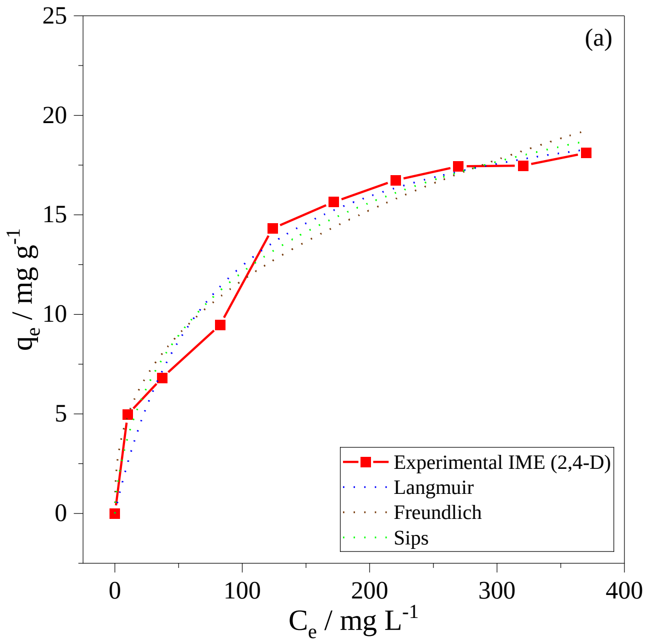

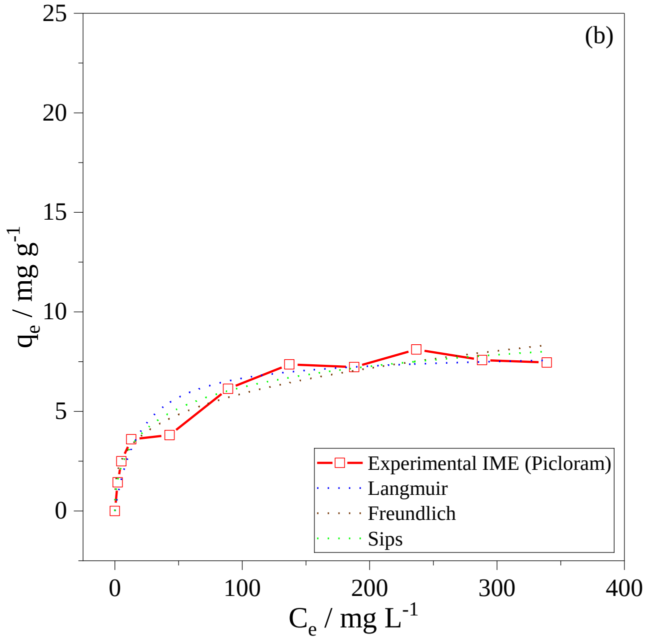

Figure S12. Fitted curves from nonlinear Langmuir, Freundlich and Sips models for2,4-D(a) and Picloram(b) adsorption on IME. Conditions: initial concentration of 2,4-D or Picloram in the renge 10-400 mg L^{-1}; 2.00 g L^{-1} of BC; 24 h; pH around 5 (no adjustment) and pH around 4 (no adjustment) for Picloram; 120 rpm; and 25.0 °C.

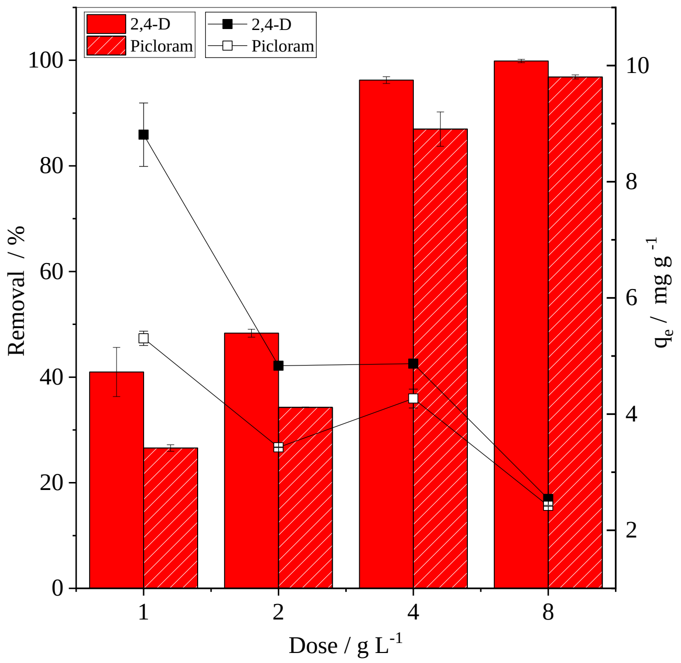

Figure S13. Effect of dose on the adsorption of 2,4-D and picloram for IME: bars indicate removal percentage and points are for q_e. Conditions: 20.0 mg L^{-1} of 2,4-D or Picloram; 24 h; pH around 5 (no adjustment) for 2,4-D and pH around 4 (no adjustment) for Picloram; 120 rpm; and 25.0 °C.

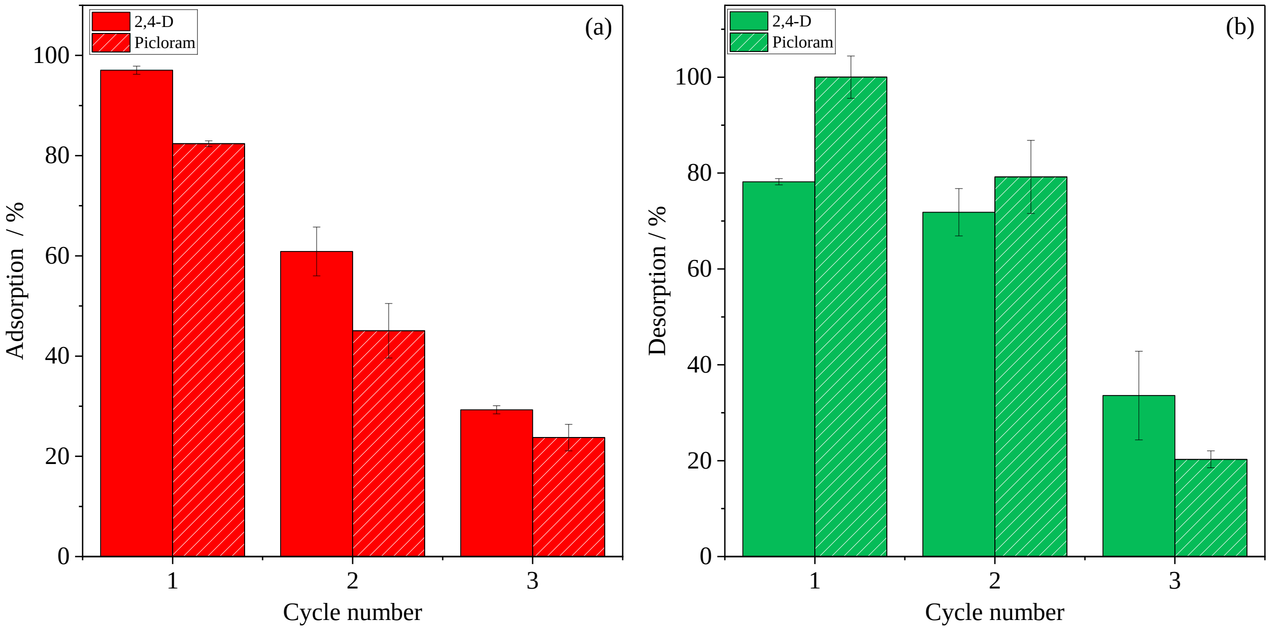

Figure S14. Studies of (a) adsorption and (b) desorption cycles of 2,4-D and Picloram on IME. Conditions: 20.0 mg L^{-1} of 2,4-D or Picloram; 24 h; pH around 5 (no adjustment) for 2,4-D and pH around 4 (no adjustment) for Picloram; 120 rpm; and 25.0 °C; Eluent for desorption: NaOH 0.01mol L^{-1}, 2 h of contact time.

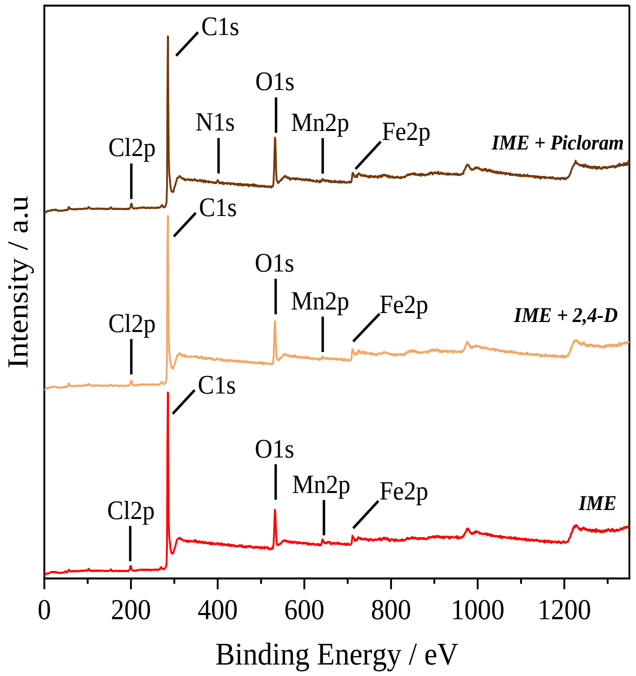

Figure S15. XPS survey spectra of IME and COP material after adsorption of 2,4-D and Picloram.

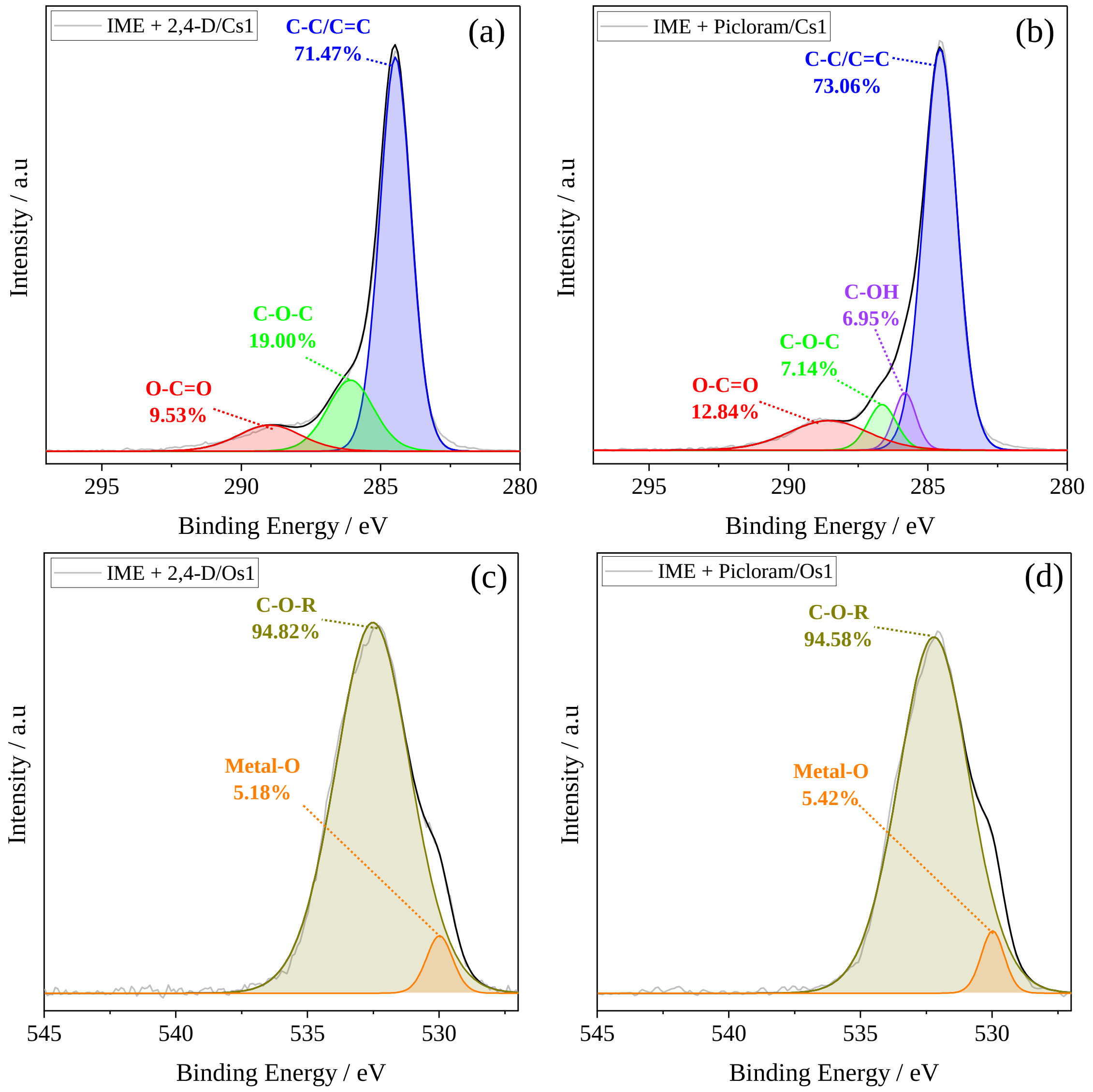

Figure S16. High-resolution C1s spectra of (a) IME + 2,4-D and (b) IME + picloram, and O1s spectra of (c) IME + 2,4-D and (d) IME + picloram after adsorption, with peak deconvolution.

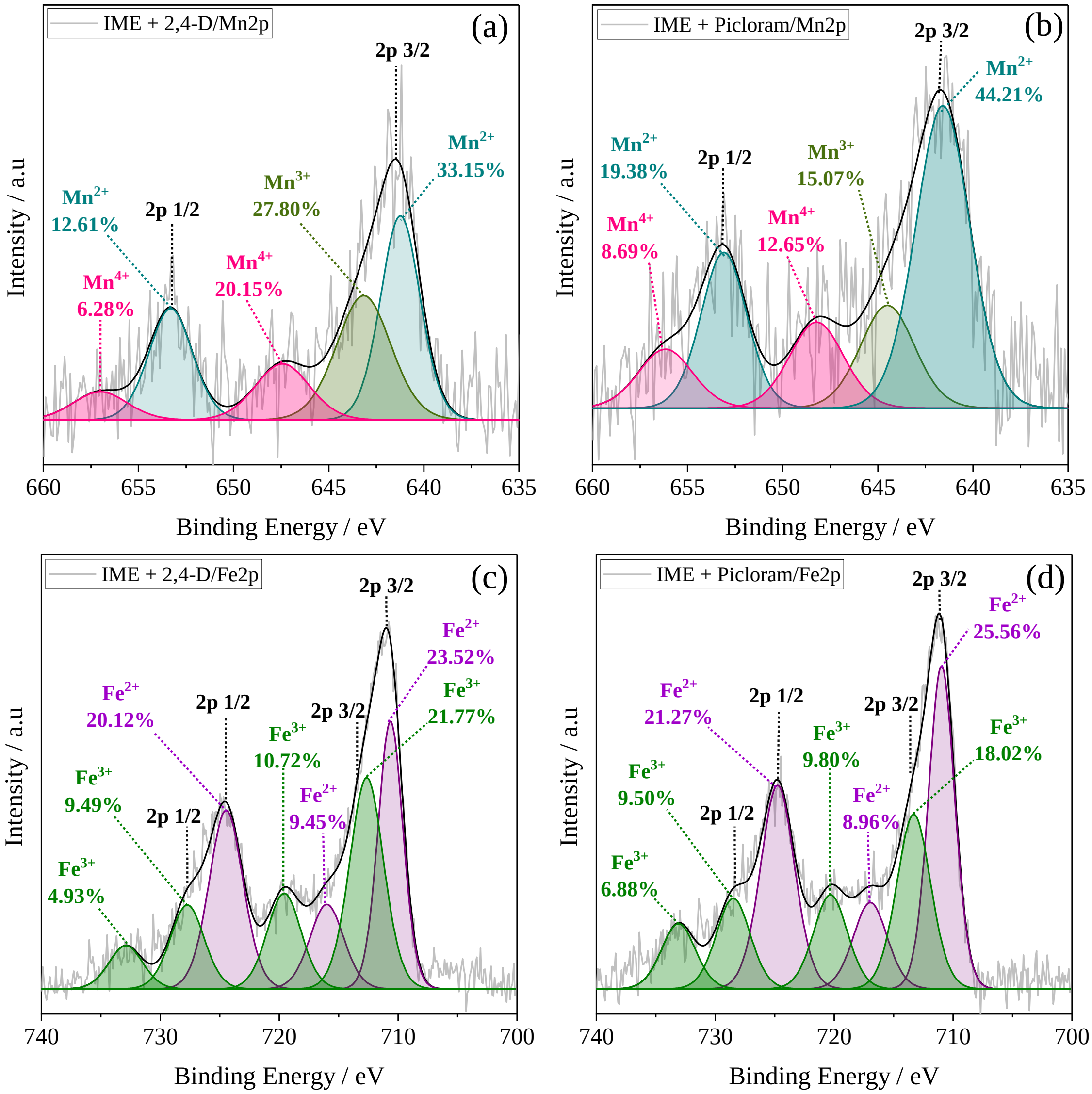

Figure S17. High-resolution Mn 2p spectra of (a) IME + 2,4-D and (b) IME + picloram, and Fe 2p spectra of (c) IME + 2,4-D and (d) IME + picloram after adsorption, with peak deconvolution.

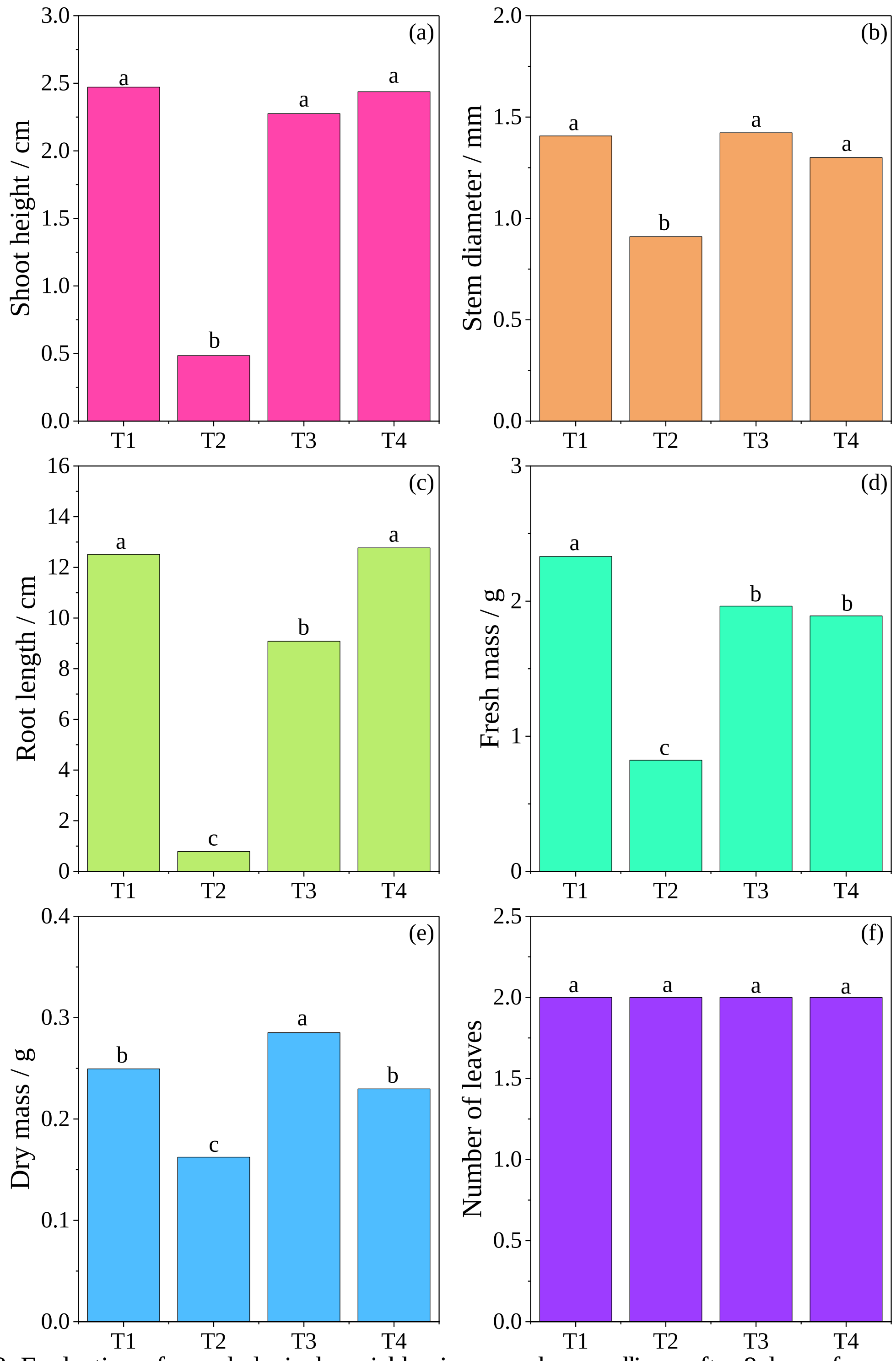

Figure S18. Evaluation of morphological variables in cucumber seedlings after 8 days of germination: (a) Shoot height, (b)Stem diameter, (c) Root length, (d) Fresh mass, (e)€ Dry mass, and (f) Number of leaves. Treatments with the same letters do not differ statistically (Tukey test, $p < 0.05$).

S2. Supplementary Tables

Table S1. Structures and properties of herbicides.

Property/Information	2,4-D	Picloram
CAS Number	94-75-7	1918-02-1
Formula	$C_8H_6Cl_2O_3$	$C_6H_3Cl_3N_2O_2$
Formula Weight	221.04 g mol^{-1}	241.46 g mol^{-1}
Solubility in Water	677 mg L^{-1}, at 25 ℃	430 mg L^{-1}, at 25 ℃
Melting Point	138.0 ℃	218.5 ℃
Dissociation Constants	$pK_a = 2.7$	$pK_a = 2.3$
Partition Coefficient	$\log K_{ow} = 2.81$	$\log K_{ow} = 0.30$
Structure	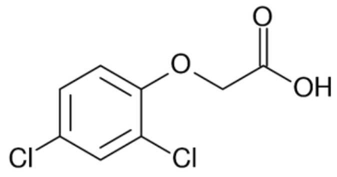	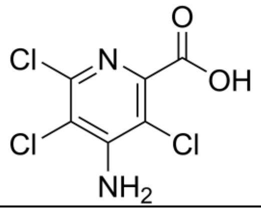

Table S2. Kinetic and isotherm model/equations used in this article.

Model	Equation	Description
Pseudo-first order	$q_{\mathrm{t}} = q_{\mathrm{e}}[1 - exp(-k_1 t)]$	q_t (mg g^{-1}): herbicide adsorbed on the adsorbent at time t (min); q_e (mg g^{-1}): adsorption amount at equilibrium; k_1 (min^{-1}): adsorption rate constant of the pseudo-first order model.
Pseudo-second order	$q_{\mathrm{t}} = \frac{k_2 {q_{\mathrm{e}}}^2 t}{1 + k_2 q_{\mathrm{e}} t}$	q_t (mg g^{-1}): herbicide adsorbed on the adsorbent at time t (min); q_e (mg g^{-1}): adsorption amount at equilibrium; k_2 (g mg^{-1} min^{-1}): adsorption rate constant of the pseudo-second order model.
Elovich	$q_{\mathrm{t}} = \frac{1}{\beta} ln(1 + \alpha\beta t)$	q_t (mg g^{-1}): herbicide adsorbed on the adsorbent at time t (min); α (mg g^{-1} min^{-1}): initial adsorption rate; β (g mg^{-1}): desorption constant.
Intraparticle diffusion model	$q_{\mathrm{t}} = k_{\mathrm{d}}\sqrt{t} + C$	q_t (mg g^{-1}): herbicide adsorbed on the adsorbent at time t (min); k_d (mg g^{-1} $min^{-1/2}$): diffusion constant; C (mg g^{-1}): constant related to the film diffusion resistance.
Langmuir	$q_{\mathrm{e}} = \frac{q_{\mathrm{max}} K_{\mathrm{L}} C_{\mathrm{e}}}{1 + K_{\mathrm{L}} C_{\mathrm{e}}}$	q_e (mg g^{-1}) and C_e (mg L^{-1}): amount of herbicide adsorbed per unit mass of adsorbent and the concentration of herbicide in solution at equilibrium, respectively; K_L (L mg^{-1}): Langmuir constant; q_{max} (mg g^{-1}): maximum amount of adsorbed herbicide.
Freundlich	$q_{\mathrm{e}} = K_{\mathrm{F}} {C_{\mathrm{e}}}^{1/n}$	q_e (mg g^{-1}) and C_e (mg L^{-1}): amount of herbicide adsorbed per unit mass of adsorbent and the concentration of herbicide in solution at equilibrium, respectively; K_F [(mg g^{-1}) (L $mg^{-1})^{1/n}$]: Freundlich constant; n: parameter related to the adsorption intensity in the Freundlich model.
Sips	$q_{\mathrm{e}} = \frac{q_{\mathrm{max}}\, K_{\mathrm{S}}\, C_{\mathrm{e}}^n}{1 + K_{\mathrm{S}}\, C_{\mathrm{e}}^n}$	q_e (mg g^{-1}) and C_e (mg L^{-1}): amount of herbicide adsorbed per unit mass of adsorbent and the concentration of herbicide in solution at equilibrium, respectively; K_S $(L\, mg^{-1})^n$: Sips constant; n: parameter to describe the heterogeneity of the surface of the adsorption system; q_{max} (mg g^{-1}): maximum amount of adsorbed herbicide.

Table S3. FTIR wavenumbers and assignments of the main bands in the spectra of the biochars.

Assignment *	Wavenumber (cm^{-1})		
	BCS	IME	COP
υC=C aromatic	1564	1564	1530
υC-H aliphatic	-	1440	-
υC-O-C ethers	1139	1139	1139
δ=C-H aromatic	873	873, 669	873
υMetal-O	-	527	564

* υ: stretch δ: angular deformation

Table S4. Yield, pH at zero charge point, and number of acidic and basic functions for the biochars.

Biochar	pH_{PZC}	Yield*	nbf	naf	n_{total} **
		%	mmol g^{-1}		
BCS	8.05	23.41	1.63	0.62	2.25
IME	4.21	40.90	1.43	3.06	4.49
COP	9.6	51.01	3.98	2.70	6.68

$*Yield\ (\%) = \frac{m_{BC}}{m_{biomass}} x100$ ** n_{total} = naf + nbf.

Table S5. Two theta and full width at half-maximum (FWHM) of crystalline peaks of IME. D represents the crystallite size.

Phase	Fe_3O_4	Fe_3O_4	MnO	Fe_3O_4	Fe_3O_4	Mn_3O_4	Fe_2O_3
2θ(°)	29.98	35.33	41.46	42.93	56.70	60.29	62.32
FWHM(°)	0.399	0.498	0.376	0.260	0.255	0.159	0.437
D(nm)	20.86	16.93	22.85	33.24	35.84	58.59	21.47

Table S6. Two theta and full width at half-maximum (FWHM) of crystalline peaks of COP. D represents the crystallite size.

Phase	Fe_3O_4	Fe_3O_4	MnO	Mn_3O_4	Fe_2O_3
2θ(°)	29.33	35.33	41.01	59.59	61.47
FWHM(°)	0.424	0.821	0.458	0.441	0.724
D(nm)	19.56	10.27	18.71	21.00	12.90

Table S7. Elemental surface composition of biochars obtained from EDS analysis.

Material	Element (weight %)								
	O	Ca	Mn	Fe	Cl	K	Al	Si	Na
BCS	73.17	13.05	-	-	0.34	9.65	-	3.79	-
IME	18.78	1.99	16.46	33.74	26.39	-	1.45	1.19	-
COP	8.65	4.28	40.64	42.11	-	-	1.17	1.05	2.11

Table S8. Elemental composition, obtained from ICP-OES, of the biochars before and after mixture with water at different pH values.

Material	Mn	Fe	Na	Mg	Al	Ca	Cu	Zn
	g kg^{-1}						mg kg^{-1}	
COP	128.3 ± 0.4	141.4 ± 0.7	5.46 ± 0.04	1.71 ± 0.01	2.17 ± 0.02	16.14 ± 0.06	28.0 ± 0.4	54.5 ± 0.7
COP - pH 2	122.7 ± 0.4	122.9 ± 0.6	0.832 ± 0.005	1.46 ± 0.01	1.07 ± 0.02	3.71 ± 0.02	16.2 ± 1.6	38.7 ± 0.2
COP - pH 7	116.6 ± 0.9	118.3 ± 1.5	2.48 ± 0.02	1.53 ± 0.01	2.237 ± 0.002	11.28 ± 0.03	27.6 ± 0.4	45.3 ± 0.2
COP - pH12	125.5 ± 0.3	139.3 ± 0.3	5.84 ± 0.03	1.739 ± 0.004	1.60 ± 0.04	16.15 ± 0.09	27.2 ± 0.3	46.3 ± 0.3
IME	20.2 ± 0.1	53.7 ± 0.1	0.166 ± 0.004	0.90 ± 0.01	1.19 ± 0.05	3.75 ± 0.02	21.4 ± 0.9	25.1 ± 0.1
IME - pH2	12.4 ± 0.1	35.8 ± 0.4	0.107 ± 0.004	0.64 ± 0.01	1.31 ± 0.02	3.73 ± 0.01	8.6 ± 1.3	25.56 ± 0.04
IME - pH7	16.1 ± 0.4	40.4 ± 0.8	0.133 ± 0.003	0.84 ± 0.01	1.39 ± 0.06	3.72 ± 0.03	11.7 ± 2.6	26.3 ± 0.1
IME - pH12	18.7 ± 0.1	51.4 ± 0.3	2.10 ± 0.02	0.89 ± 0.01	1.44 ± 0.01	3.59 ± 0.01	19.9 ± 0.7	26.5 ± 0.3
LOD (mg L^{-1})	0.001	0.003	0.02	0.0002	0.01	0.01	0.013	0.007
LOQ (mg L^{-1})	0.003	0.015	0.05	0.0006	0.02	0.04	0.039	0.021

Table S9. Atomic percentage of elements in materials before (COP and IME) and after 2,4-D and Picloram adsorption (IME) in survey spectra XPS.

Element	COP		IME		IME + 2,4-D		IME + Picloram	
	Peak	Atomic (%)	Peak	Atomic (%)	Peak	Atomic (%)	Peak	Atomic (%)
C1s	285.03	78.09	285.07	85.26	285.02	83.67	285.12	80.77
O1s	531.38	16.36	532.26	11.48	532.16	13.03	532.36	13.87
Fe2p	711.39	1.78	711.81	1.27	711.45	1.62	712.03	1.63
Mn2p	641.87	2.54	642.09	0.59	641.50	0.09	642.10	0.57
Na1s	1071.72	1.23	-	-	-	-	-	-
Cl2p	-	-	199.14	1.41	200.48	1.59	200.99	1.49
N1s	-	-	-	-	-	-	400.15	1.68

Table S10. Atomic percentage of functional groups in materials before (COP and IME) and after 2,4-D and Picloram adsorption (IME) in the C1s, O1s, Mn2p and Fe2p region XPS spectra.

Group	COP		IME		IME + 2,4-D		IME + Picloram	
	Peak	Atomic (%)	Peak	Atomic (%)	Peak	Atomic (%)	Peak	Atomic (%)
C1S								
C-C/C=C	284.5	72.44	284.5	75.30	284.5	71.47	284.6	73.06
C-OH	-	-	285.8	4.92	-	-	285.8	6.95
C-O-C	286.1	10.95	286.5	8.80	286.1	19.00	286.6	7.14
O=C-O	288.2	12.68	288.8	10.97	288.9	9.53	288.5	12.84
π-π*	291.3	3.93	-	-	-	-	-	-
O1S								
Metal-O	529.9	20.70	529.9	1.76	529.9	5.18	529.9	5.42
C=O	530.9	42.80	-	-	-	-	-	-
C-O-R	532.4	36.49	532.0	98.24	532.3	94.82	532.2	94.58
Mn2p								
2p3/2Mn^{2+}	641.1	32.73	641.1	31.31	641.2	33.15	641.6	44.21
Mn^{3+}	642.7	28.22	642.8	22.35	643.2	27.80	644.5	15.07
Mn^{4+}	646.2	7.27	646.2	19.55	647.3	20.15	648.2	12.65
2p1/2Mn^{2+}	653.1	29.03	653.4	22.85	653.3	12.61	653.0	19.38
Mn^{4+}	657.1	2.75	658.2	3.95	657.0	6.28	656.2	8.69
Fe2p								
2p3/2-Fe^{2+}	710.6	28.52	710.8	23.24	710.7	21.77	710.9	25.56
2p3/2-Fe^{3+}	713.3	17.52	713.2	20.07	712.6	23.52	713.3	18.02
Fe^{2+}	717.3	9.18	717.3	9.73	716.0	9.45	717.0	8.96
Fe^{3+}	720.3	8.53	720.4	9.39	719.6	10.72	720.3	9.80
2p1/2-Fe^{2+}	724.4	19.77	724.7	20.45	724.5	20.12	724.8	21.27
2p1/2-Fe^{3+}	727.8	8.43	728.4	9.90	727.7	9.49	728.5	9.50
Fe^{3+}	732.9	8.05	733.8	7.22	732.9	4.93	733.1	6.88

Table S11. ΔpH values of pH_{PZC}.

Initial pH	ΔpH	
	IME	COP
2.9	0.35	3.74
3.9	0.04	5.12
5.1	-0.95	3.94
6.4	-2.33	2.75
7.0	-2.08	2.34
9.8	-6.01	-0.11
11.0	-4.29	-0.28
12.0	-0.17	-0.24

Table S12. Estimated kinetic parameters for 2,4-D adsorption on IME.

Kinetic parameters[a]	2,4-D	Picloram
	IME	
$q_e(exp)^b (mg\ g^{-1})$	4.76	3.87
Pseudo-first order		
$k_1\ (min^{-1})$	1.76 x 10^{-2}	2.23 x 10^{-2}
$q_e(theo)^c (mg\ g^{-1})$	4.31	3.45
R^2	0.845	0.842
$RSS^c\ (mg^2\ g^{-2})$	3.984	2.463
AIC	–9,23	–12,46
Pseudo-second order		
$k_2\ (mg\ min^{-1})$	5.70 x 10^{-3}	8.75 x 10^{-3}
$q_e(theo)(mg\ g^{-1})$	4.57	3.67
R^2	0.922	0.921
$RSS\ (mg^2\ g^{-2})$	2.011	1.235
AIC	–17,44	–20,06
Elovich		
$\alpha\ (mg\ g^{-1}\ min^{-1})$	0.69	0.81
$\beta\ (mg\ g^{-1})$	1.57	2.05
R^2	0.978	0.979
$RSS\ (mg^2\ g^{-2})$	0.562	0.329
AIC	–32,73	–34,61
Intraparticle diffusion		
$C_1\ (mg\ g^{-1})$	1.63	1.38
$Kd_1\ (mg\ g^{-1}\ min^{-0.5})$	1.12 x 10^{-1}	1.04 x 10^{-1}
R^2	0.990	0.861
$RSS\ (mg^2\ g^{-2})$	0.060	0.050
$C_2\ (mg\ g^{-1})$	3.09	3.07
$Kd_2\ (mg\ g^{-1}\ min^{-0.5})$	4.08 x 10^{-2}	1.95 x 10^{-2}
R^2	0.887	0.879
$RSS\ (mg^2\ g^{-2})$	0.017	0.015

[a]The model parameters were obtained through nonlinear regression from equations in the Table S1. For the intraparticle diffusion model, linearization methods from equations in the Table S2. See Figure S10 and S11 for more details. [b]Values obtained at a contact time of 30 h. [c]Residual Sum of Squares.

Table S13. Estimated isothermal parameters for 2,4-D adsorption on IME.

Isothermal parameters[a]	2,4-D	Picloram
	IME	
Experimental		
q_{max} $(mg\ g^{-1})^b$	18.11	7.45
Langmuir		
K_L $(L\ mg^{-1})$	0.013	0.049
q_{max} $(mg\ g^{-1})$	22.121	8.016
R^2	0.970	0.939
RSS $(mg^2\ g^{-2})$	10.806	4.971
AIC	5.65	-2.99
Freundlich		
K_F $(mg\ g^{-1})(L\ mg^{-1})^{1/n}$	2.634	1.599
n	2.037	3.532
R^2	0.971	0.958
RSS $(mg^2\ g^{-2})$	10.600	3.418
AIC	5.47	-6.74
Sips		
q_{max} $(mg\ g^{-1})$	30.858	11.903
K_S $(L\ mg^{-1})$	0.005	0.012
n	0.663	0.519
R^2	0.977	0.968
RSS $(mg^2\ g^{-2})$	8.286	2.599
AIC	5.26	-7.47

[a]The model parameters were obtained through nonlinear regression from equations Table S2. [b] Maximum value experimentally observed. See Figure S12 for more details.
